# Effects and biological consequences of the predator-mediated apparent competition II: PDE models

**DOI:** 10.1007/s00285-025-02278-x

**Published:** 2025-10-13

**Authors:** Yuan Lou, Weirun Tao, Zhi-An Wang

**Affiliations:** 1 School of Mathematical Sciences, Shanghai Jiao Tong University, Shanghai, 200240 China; 2 School of Mathematics, Southeast University, Nanjing, 211189 China; 3 Department of Applied Mathematics, The Hong Kong Polytechnic University, Hung Hom, Hong Kong

**Keywords:** Predator-mediated, Apparent competition, Prey-taxis, Global stability, Primary 35B40, 35K57, 35Q92, 92D25

## Abstract

In Lou et al. (Lou Y, Tao W, Wang Z-A. Effects and biological consequences of the predator-mediated apparent competition I: ODE models. J. Math. Biol. 91 (2025), 47, 37 pages), the authors investigated the effects and biological consequences of the predator-mediated apparent competition using a temporal (ODE) system consisting of one predator and two prey species (one is native and the other is invasive) with Holling type I and II functional responses. This paper is a sequel to Lou et al. (Lou Y, Tao W, Wang Z-A. Effects and biological consequences of the predator-mediated apparent competition I: ODE models. J. Math. Biol. 91 (2025), 47, 37 pages.), by including spatial movements (diffusion and prey-taxis) into the ODE system and examining the spatial effects on the population dynamics under the predator-mediated apparent competition. We establish the global boundedness of solutions in a two-dimensional bounded domain with Neumann boundary conditions and the global stability of constant steady states in certain parameter regimes, by which we find a threshold dynamics in terms of the predator’s death rate. For the parameters outside the global stability regimes, we conduct a linear stability analysis to show that diffusion and/or prey-taxis can induce instability by both steady-state and Hopf bifurcations. We further use numerical simulations to illustrate that various spatial patterns are all possible, including stable spatial aggregation patterns, spatially homogeneous but time-periodic patterns, and spatially inhomogeneous and time-oscillatory patterns. It comes with a surprise that either of diffusion and prey-taxis can induce steady-state or Hopf bifurcations to generate intricate spatial patterns in the one predator-two prey system, which is sharply different from the one predator-one prey system for which neither diffusion nor prey-taxis can induce spatial patterns. These results show that spatial movements play profound roles in the emerging properties for predator-prey systems with multiple prey species. We also find that prey-taxis may play dual roles (stabilization and destabilization) and facilitate the predator-mediated apparent competition to eliminate the native prey species under the moderate initial mass of invasive prey species.

## Introduction

Predation and competition have long been a major study of interest in the field of ecology, partially because they form the foundational elements upon which complex, multispecies food webs are constructed (cf. DeAngelis 2012; Polis and Winemiller 2013; Holt and Polis 1997). Predation plays an important role in maintaining biodiversity and shaping the stable structure of ecology. For instance, in the absence of effective predator regulation, prey populations may over-reproduce and exceed the carrying capacity, leading to the depletion of smaller animal populations, the degradation of plant communities, and damage to fragile ecosystems like coral reefs (see https://blog.biodiversitylibrary.org/2012/08/why-predators-protect-biodiversity.html). Competition can happen between different species (interspecific competition) or among the same species (intraspecific competition) directly or indirectly. Among them is the predator-mediated apparent competition (cf. Holt 1977; Holt and Bonsall 2017): negative indirect interactions between prey species that are mediated by the shared natural predator. In one predator-one prey systems, predators can not drive the prey species to extinction since they would starve to death before they can find the last prey species. However, if they are fed by another prey (viewed as an invasive prey species), they may keep native prey species at a lower abundance level or even eliminate the native prey species through the predator-mediated apparent competition. For instance, populations of the damaging Pacific mites (pest) were reduced when both herbivorous Willamette mites and predatory mites were released together, which can not be achieved by releasing predatory mites alone (cf. Karban et al. 1994). Besides the above examples, apparent competition can be used in biological control (Chailleux et al. 2014; Kaser and Ode 2016; George 2017), conservation (Wittmer et al. 2013), and infectious disease ecology and invasion (Dunn et al. 2012; Strauss et al. 2012). For more applications of the predator-mediated apparent competition, we refer readers to (Chaneton and Bonsall 2000; Stige et al. 2018; Karban et al. 1994; DeCesare et al. 2010) and references therein.

Although many interspecific competitions are detectable (cf. Tilman 1987), apparent competition is difficult to detect or measure due to its indirect nature (Stige et al., 2018). Therefore, it is imperative to construct appropriate mathematical models to understand or predict the underlying qualitative or quantitative dynamics. The prototypical predator-mediated apparent competition model with *i*-species ($i\in \mathbb N_+$) was introduced by Holt in Holt (1977) and later discussed in Holt and Bonsall (2017) as follows

1.1 $$\begin{aligned} {\left\{ \begin{array}{ll} \frac{d u_i}{d t}=u_i\left[ g_i(u_i)-f_i({\textbf{u}}) w\right] ,\\ \frac{d w}{d t}=w F({\textbf{u}}), \end{array}\right. } \end{aligned}$$

where *w* and $u_i$ represent the densities of the predator and the *i*-th prey species respectively, and $\textbf{u}=(u_1,u_2,\ldots ,u_i)$. In the first equation of (1.1), the function $g_i(u_i)$ denotes the inherent per capita growth rate of the *i*-th prey species in the absence of the predator, $f_i(\textbf{u})$ is the functional response of the predator to the *i*-th prey species while $f_i({\textbf{u}}) w$ representing per capita death rate caused by predation. In the second equation of (1.1), $F({\textbf{u}})$ denotes the per capita growth rate of the predator. Under the assumption that there is no direct interspecific competition between prey species, it was found in Holt (1977) that an increase in the abundance of one prey species benefits the predator, which in turn negatively affects prey species, resulting in lower abundances for each prey. The related one predator-two prey ordinary differential systems have been studied in the literature (cf. Vance 1978; Piltz et al. 2017; Mimura and Kan-on 1986; Hsu 1981; Caswell 1978; Abrams 1999) to qualitatively explore the predator-mediated coexistence or extinction. Among others, the research of this paper is related to the following one predator-two prey model describing the predator-mediated apparent competition

1.2 $$\begin{aligned} {\left\{ \begin{array}{ll} u_{t}= u\left( 1-u / K_{1}\right) -w f_{1}(u), & t>0,\\ v_{t}= v\left( 1-v / K_{2}\right) -w f_{2}(v), & t>0,\\ w_{t}=w\left( \beta _{1} f_{1}(u)+\beta _{2} f_{2}(v)-\theta \right) ,& t>0, \end{array}\right. } \end{aligned}$$

where *u*(*t*), *v*(*t*) and *w*(*t*) represent the densities of the native prey species, the invasive prey species, and the shared predator species at time *t*, respectively. The functions $f_i$ $(i=1,2)$ and the positive parameters in (1.2) have the following biological interpretations:

- $$f_i$$ ($$i=1,2$$) - functional responses;
- $K_i$ ($i=1,2$) - carrying capacities for the prey;
- $\beta _i$ ($i=1,2$) - trophic efficiency (conversion rates);
- $\theta$ - death rate of the predator.

Based on (1.2), it is shown by the authors in Lou et al. (2025) that the predator-mediated apparent competition can indeed reduce the biomass of the native prey species. Moreover, whether the invasive prey species successfully invades or not essentially depends on its initial biomass while functional responses and other factors like the death rate of the predator may also play roles.

The effect of spatial dispersal of biological species was not considered in Lou et al. (2025). It is well-known that dispersal, an ecological process involving the spatial movement of individual/multiple species, is a strategy to increase species fitness in a heterogeneous landscape by regulating the dynamics and persistence of populations, the distribution and abundance of species as well as community structure (Hiltunen and Laakso 2013; Holt 1977; Dieckmann et al. 1999). The causes and consequences as well as the selection and evolution of dispersal strategies have been central questions in ecology extensively discussed in the literature (cf. Cosner 2014; Shurin and Allen 2001; Ron et al. 2018). Dispersal is an indispensable factor in accurately finding and predicting population dynamics in a realistic ecosystem. Usually, dispersal can be classified into two categories: undirected movement (i.e. diffusion) and directed movement (advection). Among many remarkable mathematical works, a prominent finding is that diffusion can increase population abundance in a single-species community (Lou 2006) and the slower diffuser will outcompete its faster competitor (De Mottoni 1979; Lou 2006; He and Ni 2016). The main goal of this paper is to include spatial dispersal into the ODE model (1.2) and investigate the effects of dispersal on the population dynamics in one predator-two prey systems with apparent competition mediated by predators. Since (1.2) describes the apparent competition between two prey species mediated by one predator, it is natural to consider the prey-taxis (the directed movement of a predator up a prey density gradient) apart from the random diffusion. Therefore we are motivated to consider the following PDE system:

1.3 $$\begin{aligned} {\left\{ \begin{array}{ll} u_{t}= d_1\Delta u+u\left( 1-u / K_{1}\right) -w f_{1}(u), & x\in \Omega ,\ t>0, \\ v_{t}= d_2\Delta v+v\left( 1-v / K_{2}\right) -w f_{2}(v), & x\in \Omega ,\ t>0, \\ w_{t}=d_3\Delta w-\nabla \cdot \left( w\left( \chi _{1} \nabla u+\chi _{2} \nabla v\right) \right) \\ \qquad +w\left( \beta _{1} f_{1}(u)+\beta _{2} f_{2}(v)-\theta \right) , & x\in \Omega ,\ t>0, \\ \partial _{\nu } u=\partial _{\nu } v=\partial _{\nu } w=0, & x\in \partial \Omega ,\ t>0,\\ (u,v,w)(x,0)=(u_0,v_0,w_0)(x), & x\in \Omega , \end{array}\right. } \end{aligned}$$

where the habitat $\Omega \subset \mathbb {R}^n$ ($n\ge 2$) is a bounded domain with smooth boundary $\partial \Omega$, $\partial _\nu :=\frac{\partial }{\partial \nu }$ and $\nu$ is the outward unit normal vector of $\partial \Omega$. The functions *u*(*x*, *t*), *v*(*x*, *t*) and *w*(*x*, *t*) represent the densities of the native prey species, the invasive prey species, and the shared predator at location *x* and time *t*, respectively. The functions $f_i$ and the parameters $K_i$, $\beta _i$ $(i=1,2)$ and $\theta$ have the same biological interpretations as given above. The parameters $d_1, d_2,d_3$ denote the diffusion rates of the native prey, the invasive prey, and the predator, respectively, and $\chi _i (i=1,2)$ measures the prey-taxis intensity. All the parameters in (1.3) are positive except for $\chi _1,\chi _2\ge 0$. Here we assume that both prey species move randomly within the habitat $\Omega$, while the predator undergoes random diffusion and prey-taxis. Homogeneous Neumann boundary conditions mean that no individuals can cross the boundary. In the model (1.3), the direct (i.e. interference) competition between two prey species is not considered since we focus on the predator-mediated apparent competition here. Without loss of generality, we assume that the functional responses $f_1$ and $f_2$ are of the Holling type:

1.4 $$\begin{aligned}&f_i(s)=\alpha _i s,\qquad \quad \ i=1,2,\ (\text {Holling type I}), \end{aligned}$$

1.5 $$\begin{aligned} \text {or} \qquad&f_i(s)=\frac{\gamma _i s}{1+\gamma _i h_i s},~\ i=1,2, \ (\text {Holling type II}), \end{aligned}$$

where the positive constants $\alpha _i$, $\gamma _i$ and $h_i$ $(i=1,2)$ have the following biological meanings:

- $\alpha _i$, $\gamma _i$ $(i=1,2)$ - capture rates of the predator on the prey;
- $h_i$ ($i=1,2$) - handling time spent by the predator on the prey.

The spatial predator-prey systems with diffusion involving one predator and two prey species have been considered (Chin-Chin 2019; Huang and Lin 2014; Manna et al. 2020; Feng 1993; Lakoš 1990). However, all these studies focused on direct competition between the two prey species and did not consider prey-taxis in particular. In Haskell and Bell (2020), the negative prey-taxis (the directed movement of a predator down a prey density gradient) to one prey was considered and the global existence of solutions along with pattern formation was established in one dimension. In Apreutesei et al. (2014), an optimal control problem related to the system (1.3) with $\chi _1=\chi _2=0$ is investigated to maximize the total biomass of the three species. As far as we know, the system (1.3) with (positive) prey-taxis on two prey species has not been studied in the literature. The goal of this paper is twofold: to establish the global well-posedness (existence and stability) of solutions to (1.3), and to investigate the effects of spatial movements brought to the population distribution profiles and outcome of the predator-mediated apparent competition. By studying the ODE counterpart of (1.3) in our previous work Lou et al. (2025), we find that the effects and biological consequences of predator-mediated apparent competition are a complicated ecological process depending upon many factors such as the initial mass of invasive prey species, the mortality rate of the predator and the mechanism of functional responses. In addition to the global well-posedness of (1.3), we shall further explore the following two questions. The first one is

**Q1**: Will spatial movements (diffusion or prey-taxis) facilitate or impede the effectiveness of the predator-mediated apparent competition?

It is well-known that neither diffusion nor prey-taxis can induce spatial pattern formation in one predator-one prey systems with Holling I or II functional responses (see Appendix A). Hence our second question will be

**Q2**: Will spatial movements (diffusion or prey-taxis) induce the instability to generate the spatial patterns in the one predator-two prey system?

This paper will focus on questions **Q1** and **Q2** alongside the global well-posedness of (1.3). The rest of this paper is organized as follows. Sect. 2 states the mathematical results on the global existence, boundedness, and the large time behavior of solutions to the system (1.3), as well as the biological implications concerning questions **Q1** and **Q2** based on our analytical and numerical simulations. In Sect. 3, we present the proofs for our analytical results stated in Sect. 2. The linear stability analysis is conducted in Sect. 4 to determine the parameter regimes in terms of diffusion and prey-taxis coefficients in which the steady-state or Hopf bifurcation occurs. On top of this, the numerical simulations are performed in Sect. 5 to show that either diffusion or prey-taxis can generate intricate patterns, including stable non-constant steady states, spatially inhomogeneous or heterogeneous but time-periodic patterns. In Sect. 6, we conclude our paper with a summary and discussion.

## Main results and biological implications

Our main results consist of two parts: analytical results and biological implications based on linear stability analysis and numerical simulations. They shall be readily presented below.

### Analytical results

For clarity and conciseness, we first introduce some notations used throughout the paper. Without confusion, we shall use $\int _{\Omega } f$ to denote $\int _{\Omega } f d x$. Moreover, we let

$$\begin{aligned} \begin{array}{ll} L_i:=\beta _if_i(K_i),\qquad & \lambda _i:=\frac{1}{\gamma _ih_i},\quad i=1,2,\\ L :=L_1+L_2,\quad & \theta _0:=\max \left\{ (1-\frac{\alpha _1}{\alpha _2})L_1, (1-\frac{\alpha _2}{\alpha _1})L_2 \right\} . \end{array} \end{aligned}$$

We denote the constant steady state of the system (1.3) by $E_s=\left( u_s,v_s,w_s\right)$ including extinction, predator-free, semi-coexistence and coexistence steady states, as listed in Table 1 (cf. Lou et al. 2025). To distinguish the constant coexistence steady state for different functional responses, we employ the notation

$$\begin{aligned} E_*= {\left\{ \begin{array}{ll} P_*,\qquad & \text {if }(1.4)\text { holds},\\ Q_*,& \text {if }(.1.5)\text { holds}. \end{array}\right. } \end{aligned}$$

Moreover, for the Holling type I functional response (1.4), $P_*$ is uniquely determined by

$$\begin{aligned} P_*= &  (u_*,v_*,w_*)\\ = &  \left( \frac{K_{1} \left[ (\alpha _2-\alpha _1)L_2+\alpha _1\theta \right] }{\alpha _1L_1+\alpha _2L_2}, \frac{K_{2} \left[ (\alpha _1-\alpha _2)L_1+\alpha _2\theta \right] }{\alpha _1L_1+\alpha _2L_2}, \frac{L-\theta }{\alpha _1L_1+\alpha _2L_2}\right) , \end{aligned}$$

which exists if and only if $\theta \in (\theta _0,L)$. However, the situation is more complicated for the Holling type II functional response (1.5), where the number of $Q_*$ can vary from 0 to 3 (see Lou et al. 2025).

The first result concerns the global existence and boundedness of classical solutions to (1.3), as stated in the following theorem.

#### Theorem 2.1

Let $\Omega \subset \mathbb {R}^N$ ($N\in \{1,2\}$) be a bounded domain with smooth boundary, and let $\left( u_0, v_0, w_0\right) \in \left[ W^{1, p}(\Omega )\right] ^3$ with $u_0, v_0, w_0\ge 0\ (\not \equiv 0)$ and $p>2$. Then the system (1.3) with (1.4) or (1.5) has a unique global classical solution (*u*, *v*, *w*) satisfying $u,v,w>0$ for all $t>0$, and

2.1 $$\begin{aligned} \Vert u(\cdot ,t)\Vert _{{L^\infty }(\Omega )}+\Vert v(\cdot ,t)\Vert _{{L^\infty }(\Omega )}+\Vert w(\cdot ,t)\Vert _{{L^{\infty }(\Omega )}}\le C\quad \text {for all }t>0, \end{aligned}$$

where $C>0$ is a constant independent of *t*.

#### Remark 2.1

In Theorem 2.1, the growth functions for two prey are of logistic types $g_i(s)=s(1-s/K_i)$ ($i=1,2$), the functional response $f_i$ ($i=1,2$) are of Holling type I/II, and the predator mortality rate function only includes the natural death rate. The result can indeed be extended for more general functional responses (e.g. see (Jin and Wang 2017, Theorem 1.1) with general hypotheses).

**Table 1:** Constant steady states of the system (1.3) with (1.4) or (1.5)

Type		Expression	Necessary and sufficient condition
Extinction		$E_0=(0,0,0)$	$\theta >0$
Predator-free		$E_u=(K_1,0,0),\ E_v=(0,K_2,0),\ E_{uv}=(K_1,K_2,0)$	$\theta >0$
Semi-coexistence	(1.4)	$P_1=\left( u_{P_1},0,w_{P_1}\right) =\left( \frac{\theta }{\alpha _1\beta _1}, 0,\frac{L_1-\theta }{\alpha _1L_1}\right)$	$0<\theta <L_1$
		$P_2=\left( 0,v_{P_2},w_{P_2}\right) =\left( 0,\frac{\theta }{\alpha _2\beta _2},\frac{L_2-\theta }{\alpha _2L_2}\right)$	$0<\theta <L_2$
	(1.5)	$Q_1=\left( u_{Q_1},0,w_{Q_1}\right) =\left( \frac{\theta }{(\beta _1 -h_1 \theta )\gamma _1 }, 0, \frac{\beta _1 \left( L_1 -\theta \right) }{ \gamma _1f_1(K_1)(\beta _1 - h_1 \theta )^2}\right)$	$0<\theta <L_1$
		$Q_2=\left( 0,v_{Q_2},w_{Q_2}\right) =\left( 0, \frac{\theta }{ (\beta _2 - h_2 \theta )\gamma _2}, \frac{\beta _2 \left( L_2-\theta \right) }{ \gamma _2 f_2(K_2) (\beta _2 - h_2 \theta )^2}\right)$	$0<\theta <L_2$
Coexistence	(1.4)	$P_*$	$\theta _0<\theta <L$
	(1.5)	$Q_*$	Unclear$^*$

Note: $^*$For the existence of $Q_*$, it is hard to determine the necessary and sufficient conditions, and $0<\theta <L$ is a necessary but not sufficient condition (see (Lou et al. 2025, Remark 2.1) for details)

We next give the global stability of the positive constant steady state $E_*=\left( u_*,v_*,w_*\right)$ for $0<\theta < L$, and the predator-free constant steady state $E_{uv}$ for $\theta \ge L$. If $E_*$ exists, we define the positive constant

2.2 $$\begin{aligned} d_* := {\left\{ \begin{array}{ll} \displaystyle \frac{w_*}{4}\left( \frac{\alpha _1\chi _1^2 K_1^2}{\beta _1 d_1f_1(u_*)}+\frac{\alpha _2\chi _2^2 K_2^2 }{\beta _2 d_2f_2(v_*)}\right) ,& \quad \text {if }(1.4)\text { holds},\\ \displaystyle \frac{w_*}{4}\left( \frac{\gamma _1\chi _1^2 K_1^2}{\beta _1 d_1f_1(u_*)}+\frac{\gamma _2\chi _2^2 K_2^2 }{\beta _2 d_2f_2(v_*)}\right) ,& \quad \text {if }(1.5)\text { holds}. \end{array}\right. } \end{aligned}$$

The global stability results are stated below.

#### Theorem 2.2

(Global stability for Holling type I) Let the assumptions in Theorem 2.1 hold with $f_i (i=1,2)$ given by (1.4). Let (*u*, *v*, *w*) be the solution of (1.3) obtained in Theorem 2.1 and $d_*$ be given by (2.2). Then the following global stability results hold.

(i) If $\theta \in (\theta _0,L)$, then the unique positive constant steady state $P_*$ is globally asymptotically stable if $d_3\ge d_*$, where “=" holds only if $\Vert u_0\Vert _{L^{\infty }(\Omega )}\le K_1$ and $\Vert v_0\Vert _{L^{\infty }(\Omega )}\le K_2$.
(ii) If $\theta \ge L$, then the predator-free constant steady state $E_{uv}$ is globally asymptotically stable.

#### Theorem 2.3

(Global stability for Holling type II) Assume the assumptions in Theorem 2.1 hold with $f_i (i=1,2)$ given by (1.5). Let (*u*, *v*, *w*) be the solution of (1.3) obtained in Theorem 2.1 and $d_*$ be given by (2.2). Then the following global stability results hold.

(i) If $\theta \in (0,L)$, and $Q_*=\left( u_*,v_*,w_*\right)$ is a positive constant steady state (if exists) satisfying
  2.3 $$\begin{aligned} (K_1,K_2)\in \Lambda _*:=\left\{ (K_1,K_2)\ \bigg |\ K_1\le \lambda _1+u_*,\ K_2\le \lambda _2+v_*\right\} , \end{aligned}$$
  then $Q_*$ is globally asymptotically stable if $d_3\ge d_*$, where “=" holds only if $\Vert u_0\Vert _{L^{\infty }(\Omega )}\le K_1$ and $\Vert v_0\Vert _{L^{\infty }(\Omega )}\le K_2$.
(ii) If $\theta \ge L$, then the predator-free constant steady state $E_{uv}$ is globally asymptotically stable.

### Biological implications based on numerical simulations

Now we shall address questions raised in Q1 and Q2 by using linear stability analysis and numerical simulations.

By viewing one prey *u* and the predator *w* as the native species, and the other prey *v* as the invasive one in (1.3), we consider Holling type I and II functional responses and establish the global stability of the coexistence (i.e. positive) and the predator-free constant steady states in Theorem 2.2 and Theorem 2.3, respectively. By these results, we find threshold dynamics in terms of the predator’s death/mortality rate. To investigate the spatial pattern formation, we conduct linear instability analysis outside the parameter regimes of global stability given in Theorem 2.2 and Theorem 2.3 (see Sect. 4) and perform numerical simulations (see Sect. 5) for the system (1.3) with (1.4) or (1.5) to pinpoint the possible biological consequences resulting from the predator-mediated apparent competition. Our main findings are summarized below.

**Fig. 1:**
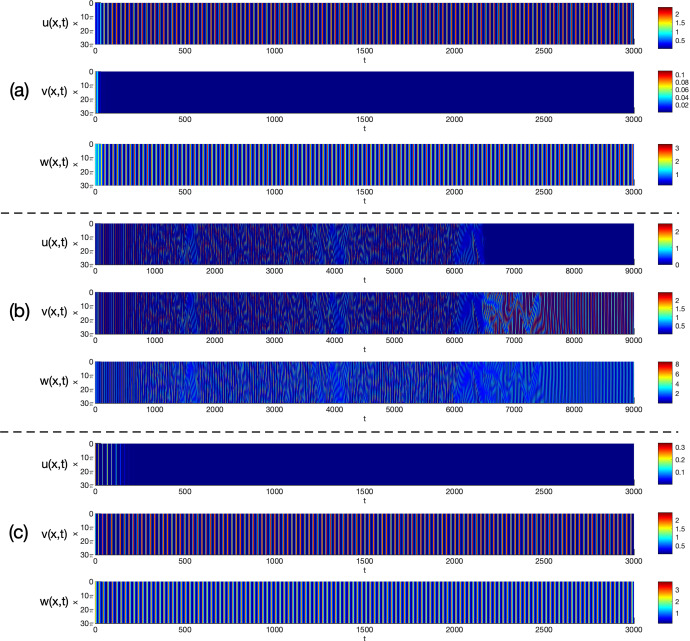
Numerical simulations for the system (1.3) with (1.5) in the interval $\Omega =(0,30\pi )$, under the parameter setting (4.1), $\theta =\frac{1}{4}$, $d_1=d_2=d_3=1$ and $(\chi _1,\chi _2)=(2,1)$. The initial data are given by (5.1) with $R=0.1$ in **(a)**, $R=0.36$ in **(b)**, and $R=1$ in **(c)**

**Fig. 2:**
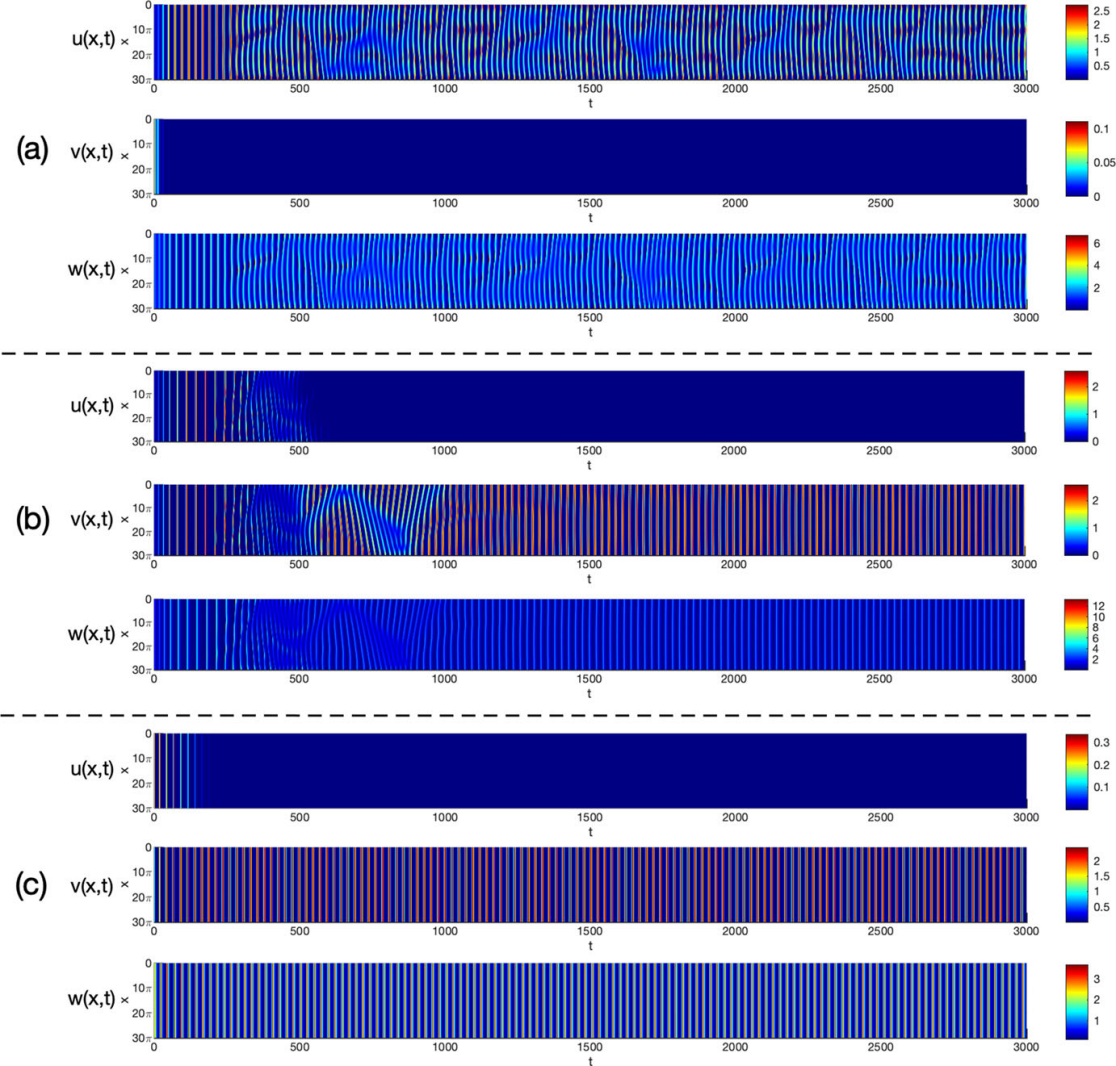
Numerical simulations for the system (1.3) with (1.5) in the interval $\Omega =(0,30\pi )$, under the parameter setting (4.1), $\theta =\frac{1}{4}$, $d_1=d_2=d_3=1$ and $(\chi _1,\chi _2)=(10,1)$. The initial data are given by (5.1) with $R=0.1$ in **(a)**, $R=0.36$ in **(b)**, and $R=1$ in **(c)**

**Fig. 3:**
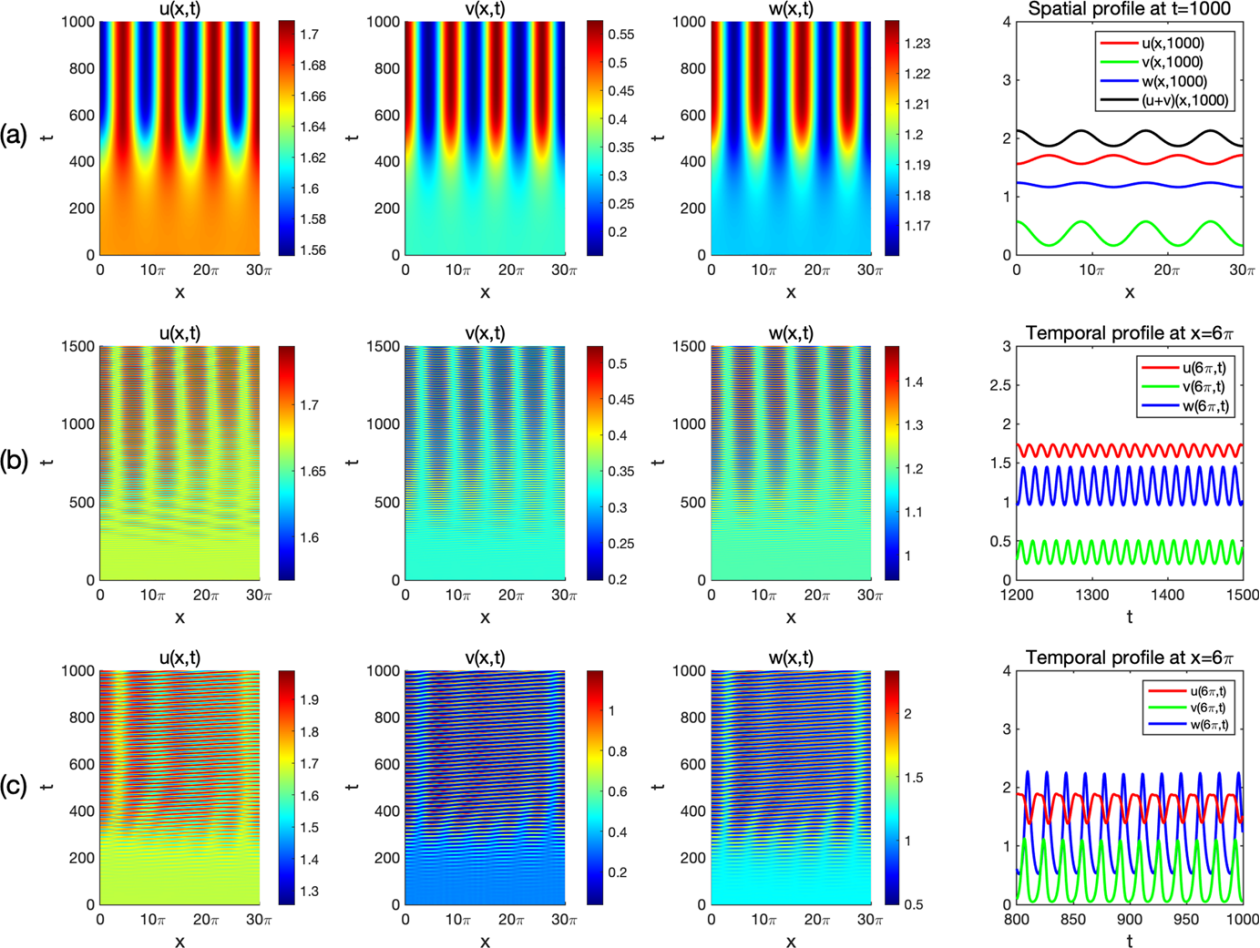
Numerical simulations for the system (1.3) with (1.5) in the interval $\Omega =(0,30\pi )$, under the parameters setting (4.15) with $\chi _1=\chi _2=0$, for different values of $(d_1,d_2,d_3)$ given in (5.2): **E.1** in row **(a)**, **E.2** in row **(b)**, and **E.3** in row **(c)**. The initial value is given as a small random perturbation of ${Q_*^1}=\left( \frac{5}{3},\frac{1}{3},\frac{32}{27}\right)$

**Fig. 4:**
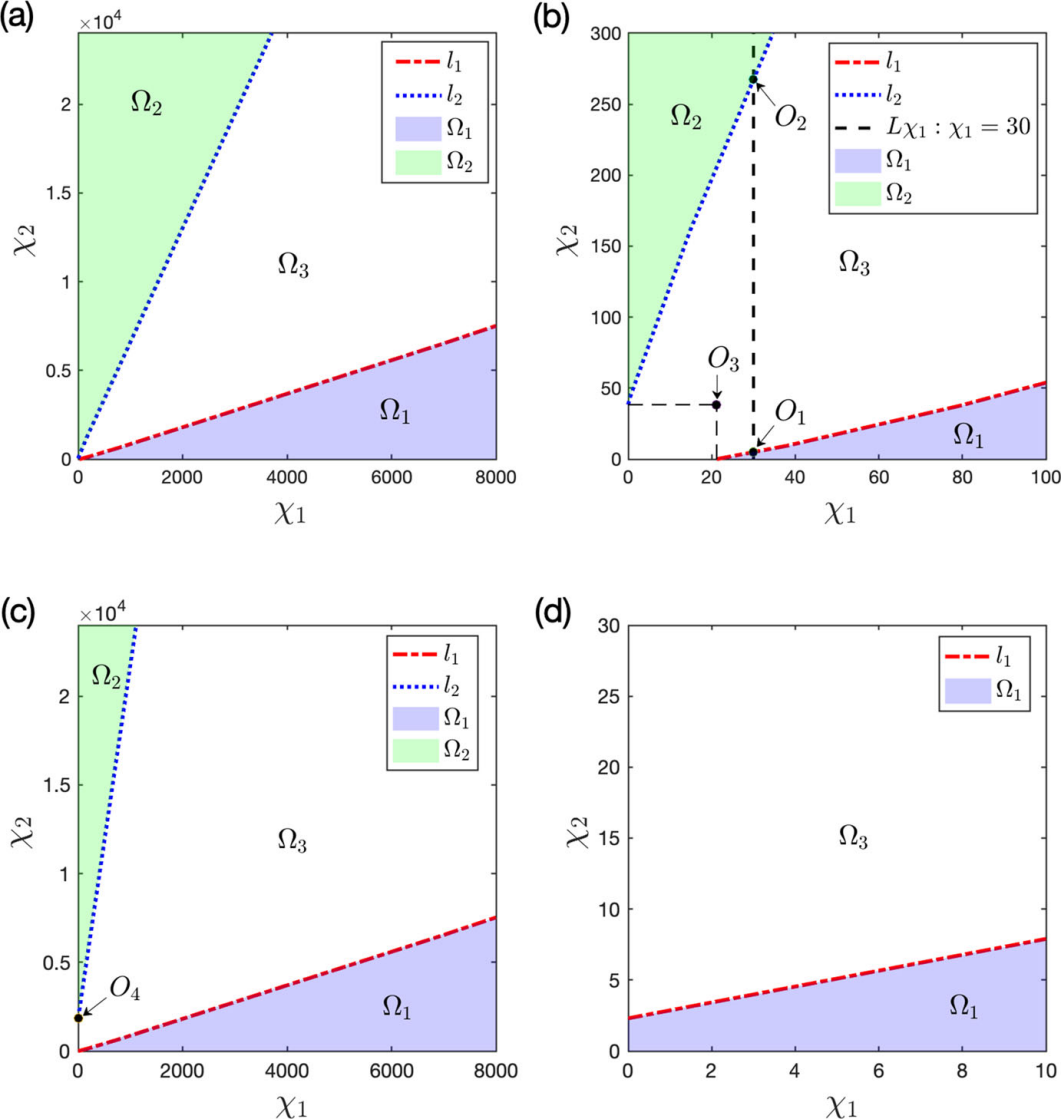
Stability parameter regions $\Omega _i$ ($i=1,2,3$) in the $\chi _1$-$\chi _2$ plane for $d_1=d_2=1$ and different values of $d_3$, with $d_3=1$ in **(a)-(b)** and $d_3=60$ in **(c)-(d)**, where figures in **(b)** and **(d)** are the amplified portions of **(a)** and **(c)** near the origin, respectively. The auxiliary line $L\chi _1$ in **(b)** intersects with ${ l_1}$ and ${ l_2}$ at the points $O_1\approx (30,4.9275)$ and $O_2\approx (30,267.3756)$, respectively, $O_3\approx (21.1921,38.34)$ and $O_4\approx (0,1839.6170)$

**Fig. 5:**
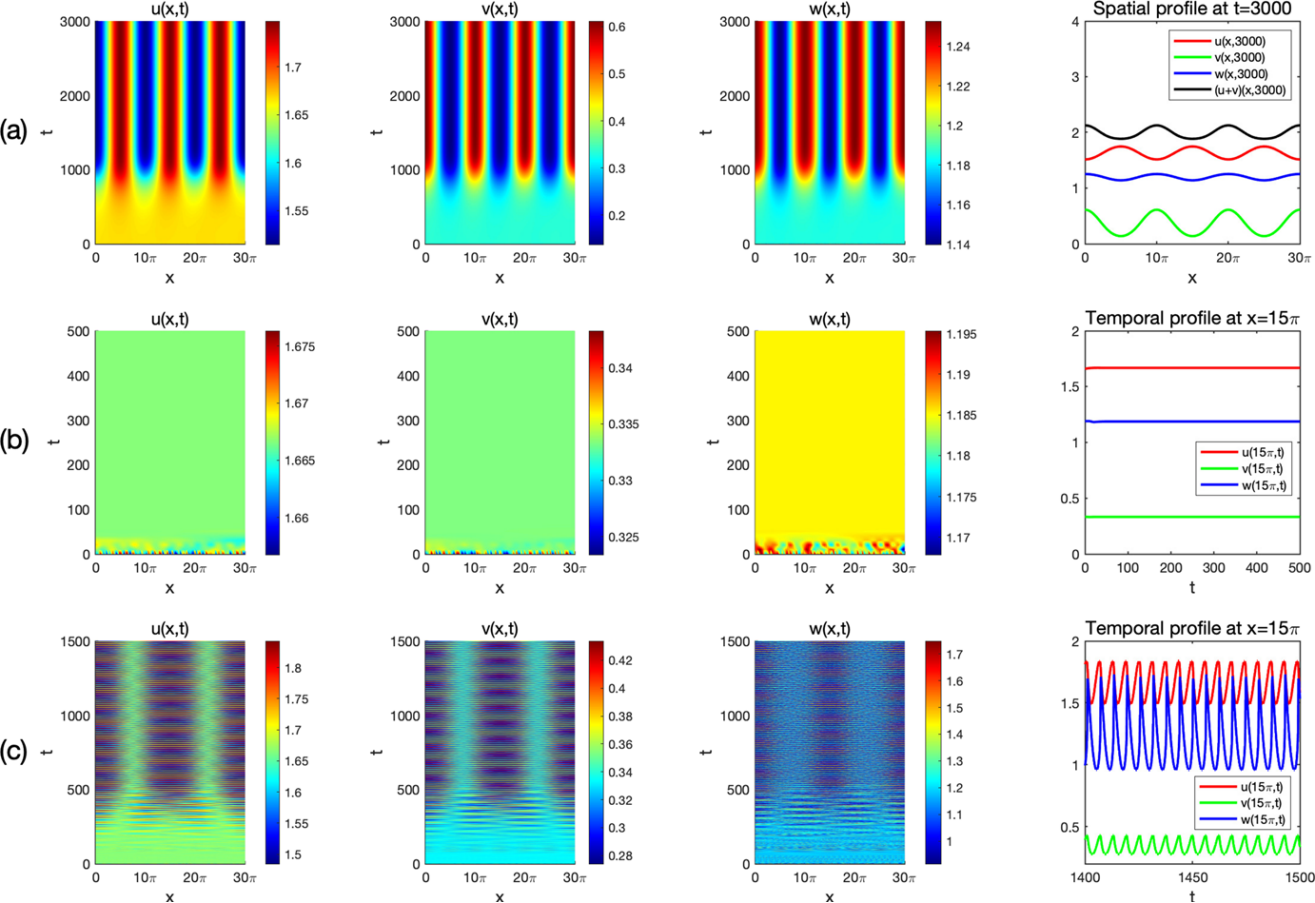
Numerical simulations for the system (1.3) with (1.5) in the interval $\Omega =(0,30\pi )$, under the parameter setting (5.6), $\chi _1=30$ and different values of $\chi _2$: $\chi _2=3$ in **(a)**, $\chi _2=100$ in **(b)**, and $\chi _2=500$ in **(c)**. The initial value is chosen as a small random perturbation of ${Q_*^1}=\left( \frac{5}{3},\frac{1}{3},\frac{32}{27}\right)$

1. For the Holling type I functional response (1.4), diffusion or prey-taxis can not destabilize system (1.3) to generate spatial patterns and the long-time dynamics of (1.3) are mainly determined by the reaction terms, similar to its ODE counterpart.
2. For the Holling type II functional response (1.5), the spatial movements can have profound effects on the population dynamics of system (1.3). For the case of symmetric apparent competition (namely, the two prey species have the same ecological characteristics), we find the initial biomass of invasive prey species is crucial to determine whether the invasion is successful: small invasion biomass leads to failed invasion, moderate invasion biomass leads to successful invasion and species coexistence, while large invasion biomass results in successful invasion which further wipes out the native prey species (see Fig. 1). By comparing the numerical results shown in Fig. 1-(b) and Fig. 2-(b), we find that strong prey-taxis for the native prey species can help the invasive species outcompete the native prey species (i.e. eliminate the native prey species) through the predator-mediated apparent competition given the success of invasion with moderate initial invasion biomass. This implies that prey-taxis can help the invasive prey species wipe out the native prey species with moderate invasion biomass only through the predator-mediated apparent competition.
3. It is known that neither diffusion nor prey-taxis can destabilize one predator-one prey systems (see Appendix A) with Holling type I or II functional responses to promote the spatial pattern formations. However, for the one predator-two prey system (1.3) with the Holling type II functional response, we find that either diffusion or prey-taxis can destabilize positive uniform equilibria (see Table 6 and Fig. 4) to produce intricate spatial patterns (see Figs. 3 and 5). This finding entails that the effect of diffusion or prey-taxis may be substantially different as the number of prey species increases. Namely, spatial movements may play more profound roles in predator-prey systems with more prey species.
4. In the one predator-two prey spatial system (1.3) with the Holling type II functional response, either diffusion or prey-taxis can destabilize positive equilibria to generate spatial patterns in some parameter regimes (see Remark 5.3). However, the prey-taxis may also stabilize (or homogenize) the system in other parameter regimes (see Figs. 4 and 5). Therefore the prey-taxis have different effects (stabilization and destabilization) depending on the suitable values of $\chi _1$ and $\chi _2$ (i.e. the intensity of prey-taxis), indicating that spatial movements may play complex roles in predator-prey systems consisting of multiple prey species.

In this paper we attempt to understand the structures and functions of predator-prey systems with apparent competition and spatial movement. To summarize, our studies reveal some emerging properties of predator-mediated apparent competition models with movement: First, for symmetric apparent competition there exist critical thresholds for the initial biomass of the invasive prey species to determine whether the invasion is successful, and whether it will wipe out the native prey species. Second, spatial movement can induce spatiotemporal patterns in one predator-two prey system, but not for one predator-one prey system. It will be of interest to further investigate the functions of these spatiotemporal patterns, making the connections between the structures of these ecological systems and the functions of such structures.

## Global boundedness and stability

### Global boundedness

We first prove Theorem 2.1 concerning the global existence and boundedness of solutions to the system (1.3) with either (1.4) or (1.5). The local existence of solutions can be established by using Amann’s theorem (cf. Amann 1990, 1993). Additionally, the approach developed for one predator-one prey systems with prey-taxis in Jin and Wang (2017) can be adapted here to prove Theorem 2.1 with slight adjustments.

#### Lemma 3.1

Let the assumptions in Theorem 2.1 hold. Then there exists some $T_{max} \in (0, \infty ]$ such that the system (1.3) with (1.4) or (1.5) admits a unique classical solution

$$\begin{aligned} (u, v, w) \in \left[ C^0\left( \bar{\Omega } \times \left[ 0, T_{max }\right) \right) \cap C^{2,1}\left( \bar{\Omega } \times \left( 0, T_{max }\right) \right) \right] ^3 \end{aligned}$$

satisfying $u, v, w>0$ for all $t>0$. Moreover, we have

3.1 $$\begin{aligned} \text {either}\quad T_{max} = \infty ,\quad \text {or}\quad \limsup _{t \nearrow T_{max }} \Vert w (\cdot , t)\Vert _{L^\infty (\Omega )}=\infty . \end{aligned}$$

#### Lemma 3.2

Let the assumptions in Theorem 2.1 hold. Then for all $t\in (0, T_{max})$, the solution of the system (1.3) with (1.4) or (1.5) satisfies

3.2 $$\begin{aligned} u\le M_1:=\max \left\{ \Vert u_0\Vert _{L^{\infty }(\Omega )},K_1\right\} , \quad v\le M_2:=\max \left\{ \Vert v_0\Vert _{L^{\infty }(\Omega )},K_2\right\} , \end{aligned}$$

and

$$\begin{aligned} \Vert w\Vert _{L^{1}(\Omega )} \le M_3&:=\max \left\{ \beta _1\Vert u_0\Vert _{L^{1}(\Omega )}+\beta _2\Vert v_0\Vert _{L^{1}(\Omega )}+\Vert w_0\Vert _{L^{1}(\Omega )}, \right. \\&\quad \left. \frac{(1+\theta )^2}{4\theta }\left( \beta _1K_1+\beta _2K_2\right) |\Omega |\right\} . \end{aligned}$$

#### Proof

Note that (3.2) is a direct consequence of (Jin and Wang 2017, Lemma 2.2) based on a comparison principle. We next prove the boundedness of $\Vert w\Vert _{L^{1}(\Omega )}$. Let

$$\begin{aligned} z(x,t):=\beta _1 u(x,t)+\beta _2 v(x,t)+w(x,t)\quad \text {for all }(x,t)\in \Omega \times (0, T_{max}). \end{aligned}$$

Then using (1.3), integrating by parts, and Young’s inequality, we have

$$\begin{aligned} \frac{d}{dt}\int _\Omega z =&~ \beta _1\int _\Omega \left( u-\frac{u^2}{K_1}\right) +\beta _2\int _\Omega \left( v-\frac{v^2}{K_2}\right) -\theta \int _\Omega w\\ \le&~ \beta _1\int _\Omega \left[ -\theta u+\frac{K_1(1+\theta )^2}{4}\right] +\beta _2\int _\Omega \left[ -\theta v+\frac{K_2(1+\theta )^2}{4}\right] -\theta \int _\Omega w\\ \le&~ -\theta \int _\Omega z+\frac{(1+\theta )^2}{4}\left( \beta _1K_1+\beta _2K_2\right) |\Omega |\quad \text {for all }t\in (0, T_{max}). \end{aligned}$$

An application of Grönwall’s inequalitygives $\Vert z\Vert _{L^{1}(\Omega )}\le M_3$, which along with the non-negativity of the solution completes the proof. $\square$

#### Lemma 3.3

Let the assumptions in Theorem 2.1 hold. Then the solution to the system (1.3) with (1.4) or (1.5) satisfies

3.3 $$\begin{aligned} \Vert u(\cdot ,t)\Vert _{{L^\infty }(\Omega )}+\Vert v(\cdot ,t)\Vert _{{L^\infty }(\Omega )}+\Vert w(\cdot ,t)\Vert _{L^{\infty }(\Omega )}\le C\quad \text {for all }t\in (0, T_{max}), \end{aligned}$$

where *C* is a positive constant independent of *t*.

#### Proof

Based on Lemma 3.2, (3.3) can be established by similar arguments as in (Jin and Wang 2017, section 3) for one predator-one prey systems with prey-taxis. Here the two prey species bring no essential difficulty in deriving (3.3) since there is no direct interaction between the two prey species. The routine procedures are omitted for brevity. $\square$

#### Proof of Theorem 2.1

It follows immediately from (3.1) and (3.3) that $T_{max}=\infty$, which alongside (3.3) gives (2.1). The proof is completed. $\square$

### Global stability

This subsection is devoted to proving Theorem 2.2 and Theorem 2.3. Note that the constant steady states $E_0$, $E_u$ and $E_v$ are saddles, and $E_{uv}$ is also a saddle for $\theta \in (0,L)$ (see Proposition 4.1). In this section, we shall construct appropriate Lyapunov functions to investigate the global stability of positive constant steady states for $0<\theta <L$ and the predator-free constant steady state $E_{uv}$ for $\theta \ge L$. We first give the following higher-order regularities of the solution to the system (1.3) with (1.4) or (1.5).

#### Lemma 3.4

Let the assumptions in Theorem 2.1 hold, and let (*u*, *v*, *w*) be the solution of the system (1.3) with (1.4) or (1.5). Then for any given $\sigma \in (0,1)$, there exists a positive constant *C* such that

$$\begin{aligned} &  \Vert u\Vert _{C^{2+\sigma , 1+\frac{\sigma }{2}}{\left( \bar{\Omega } \times [1,\infty )\right) }}+ \Vert v\Vert _{C^{2+\sigma , 1+\frac{\sigma }{2}}{\left( \bar{\Omega } \times [1,\infty )\right) }}\\ &  \quad + \Vert w \Vert _{C^{2+\sigma , 1+\frac{\sigma }{2}}{\left( \bar{\Omega } \times [1,\infty )\right) }}\le C. \end{aligned}$$

#### Proof

With (2.1), the conclusion can be proved by a bootstrap argument based on the $L^{p}$-estimate and the Schauder estimate (cf. Gary 1996). We omit the details for brevity. $\square$

The following auxiliary lemma will be used to prove the global stability.

#### Lemma 3.5

(Wang 2018, Lemma 1.1) Let $a \ge 0$ and $b>0$ be two constants, $F(t) \ge 0, \int _{a}^{\infty } H(t) \textrm{d} t<\infty$. Assume that $E \in C^{1}([a, \infty ))$ is bounded from below and satisfies

$$\begin{aligned} E^{\prime }(t) \le -b F(t)+ H(t) \quad \text{ in } [a, \infty ). \end{aligned}$$

If $F \in C^{1}([a, \infty ))$ and $F^{\prime }(t) \le C$ in $[a, \infty )$ for some constant $C>0$, or $F \in C^{\theta }([a, \infty ))$ and $\Vert F\Vert _{C^{\theta }([a, \infty ))} \le C$ for some constants $0<\theta <1$ and $C>0$, then $\lim \limits _{t \rightarrow \infty } F(t)=0$.

For $t>0$ and a constant steady state $E_s=(u_s,v_s,w_s)$, we let

3.4 $$\begin{aligned} {\mathcal {E}}(t;E_s):= &  \Gamma _1\int _\Omega \left( u-u_s-u_s\ln \frac{u}{u_s}\right) +\Gamma _2\int _\Omega \left( v-v_s-v_s\ln \frac{v}{v_s}\right) \nonumber \\ &  +\int _\Omega \left( w-w_s-w_s\ln \frac{w}{w_s}\right) , \end{aligned}$$

where the positive constants $\Gamma _1$ and $\Gamma _2$ are given by

3.5 $$\begin{aligned} \Gamma _i:= {\left\{ \begin{array}{ll} \frac{\beta _i f_i(u_s)}{\alpha _i u_s} =\beta _i,\quad & \text {if }(1.4)\text { holds},\\ \frac{\beta _i f_i(u_s)}{\gamma _i u_s} =\frac{\beta _i}{1+\gamma _ih_iu_s},\quad & \text {if }(1.5)\text { holds}, \end{array}\right. } \qquad i=1,2. \end{aligned}$$

Then we can prove the global stability of $E_{uv}$ and the positive constant steady states, as to be shown below.

### Global stability for $\theta \ge L$

The predator-free constant steady state $E_{uv}$ of the system (1.3) with (1.4) or (1.5) is globally asymptotically stable whenever $\theta \ge L$.

#### Lemma 3.6

[Global stability of $E_{uv}$] Let the assumptions in Theorem 2.1 hold, and let $\theta \ge L$. Then for any $\sigma \in (0,1)$, the solution (*u*, *v*, *w*) of the system (1.3) with (1.4) or (1.5) satisfies

$$\begin{aligned} \lim _{t \rightarrow \infty }\left( \Vert u-K_1\Vert _{C^{2+\sigma }(\bar{\Omega })}+\Vert v-K_2\Vert _{C^{2+\sigma }(\bar{\Omega })}+\Vert w\Vert _{C^{2+\sigma }(\bar{\Omega })}\right) =0. \end{aligned}$$

#### Proof

Let $E_s=E_{uv}=(K_1,K_2,0)$ in (3.4) and (3.5). Then using (1.3), (3.4), (3.5), and integrating by parts, for all $t>0$, one has

3.6 $$\begin{aligned} {\mathcal {E}}' (t;E_{uv}) =&~ \underbrace{-\Gamma _1d_1K_1\int _\Omega \frac{|\nabla u|^2}{u^2}-\Gamma _2d_2K_2\int _\Omega \frac{|\nabla v|^2}{v^2}}_{\le 0} \nonumber \\&\quad +\int _\Omega w\left( \beta _1 f_1(u)+\beta _2 f_2(v)-\theta \right) \nonumber \\&\quad +\Gamma _1 \int _\Omega \left( 1-\frac{u}{K_1}-\frac{wf_1(u)}{u}\right) \left( u-K_1\right) \nonumber \\&\quad +\Gamma _2 \int _\Omega \left( 1-\frac{v}{K_2}-\frac{wf_2(v)}{v}\right) \left( v-K_2\right) . \end{aligned}$$

The combination of (1.4), (1.5), (3.5) and $\theta \ge L=\beta _1f_1(K_1)+\beta _2f_2(K_2)$ indicates that

3.7 $$\begin{aligned} w\left( \beta _1 f_1(u)+\beta _2 f_2(v)-\theta \right) \le&~ \beta _1 w\left( f_1(u)-f_1(K_1)\right) +\beta _2 w \left( f_2(v)-f_2(K_2)\right) \nonumber \\ =&~ \Gamma _1 \frac{wf_1(u)}{u}(u-K_1)+\Gamma _2 \frac{wf_2(v)}{v}(v-K_2) \nonumber \\&\qquad \quad \text {for all }t>0, \end{aligned}$$

where we have used the fact that $\beta _i(f_i(s)-f_i(K_i))=\Gamma _i\frac{f_i(s)}{s} (s-K_i)$ for $s>0$, $i=1,2$, since

3.8 $$\begin{aligned} \beta _i(f_i(s)-f_i(K_i))= {\left\{ \begin{array}{ll} \alpha _i\beta _i(s-K_i)=\Gamma _i\frac{f_i(s)}{s}(s-K_i),\quad & \text {if }(1.4)\text { holds},\\ \frac{\gamma _i\beta _i(s-K_i)}{(1+\gamma _ih_iK_i)(1+\gamma _ih_is)} =\Gamma _i\frac{f_i(s)}{s} (s-K_i),\quad & \text {if }(1.5)\text { holds}. \end{array}\right. } \end{aligned}$$

Substituting (3.7) into (3.6), we obtain

3.9 $$\begin{aligned} {\mathcal {E}}' (t;E_{uv})\le -\frac{\Gamma _1}{K_1} \int _\Omega (u-K_1)^2-\frac{\Gamma _2}{K_2} \int _\Omega (v-K_2)^2=:-\mathcal F_1(t)\quad \text {for all }t>0. \end{aligned}$$

For any constant ${s_*}>0$, the function $\phi _1(s):=s-{s_*}-{s_*}\ln \frac{s}{{s_*}}$ satisfies $\phi _1(s)\ge \phi _1({s_*})=0$ for $s>0$, and $\phi _1(s)=0$ if and only if $s={s_*}$. Therefore, it holds that

$$\begin{aligned} {\mathcal {E}} (t;E_{uv})\ge 0\quad \text {for all }t>0. \end{aligned}$$

This alongside Lemma 3.4 and Lemma 3.5 gives $\lim \limits _{t \rightarrow \infty } {\mathcal {F}}_1(t)=0$, namely

$$\begin{aligned} \lim _{t \rightarrow \infty } (\Vert u-K_1\Vert _{L^{2}(\Omega )}+\Vert v-K_2\Vert _{L^{2}(\Omega )})=0. \end{aligned}$$

Take $\tilde{\sigma }\in (0,1)$ satisfying $0<\sigma<{\tilde{\sigma }}<1$. Then Lemma 3.4 implies that $u(\cdot , t)$ and $v(\cdot , t)$ are bounded in $C^{2+\tilde{\sigma }}(\bar{\Omega })$ for $t \ge 1$ (since the embedding $C^{2+\tilde{\sigma }}\left( \bar{\Omega } \right) \hookrightarrow C^{2+\sigma }\left( \bar{\Omega } \right)$ is compact thanks to the Ascoli-Arzelà theorem). Using the compact arguments and uniqueness of limits (cf. (Bei 2011, Proof of Theorem 6.1 and Remark 6.1) for instance) we have

3.10 $$\begin{aligned} \lim _{t \rightarrow \infty }\left( \Vert u-K_1\Vert _{C^{2+\sigma }(\bar{\Omega })}+\Vert v-K_2\Vert _{C^{2+\sigma }(\bar{\Omega })}\right) =0 \end{aligned}$$

holds for any $\sigma \in (0,1)$. It remains to prove

3.11 $$\begin{aligned} \lim _{t \rightarrow \infty }\Vert w\Vert _{C^{2+\sigma }(\bar{\Omega })}=0. \end{aligned}$$

Define $\bar{g}(t)=\frac{1}{|\Omega |} \int _{\Omega } g(x,t) \mathrm {~d} x$. Then by the first equation of (1.3), we have

3.12 $$\begin{aligned} {\bar{u}}'(t)=\frac{d}{dt}\left( \frac{1}{|\Omega |}\int _\Omega u\right) =\frac{1}{|\Omega |}\int _\Omega u\left( 1-\frac{u}{K_1}\right) -\frac{1}{|\Omega |}\int _\Omega {wf_1(u)}=:I_1(t)+I_2(t).\nonumber \\ \end{aligned}$$

Clearly, (3.10) implies $I_1(t) \rightarrow 0$ as $t \rightarrow \infty$.

Lemma 3.4 implies $\Vert \bar{u}^{\prime }\Vert _{C^{\sigma / 2}([1, \infty ))} \le C$, which means that there exists a constant $C_\sigma >0$ such that $|\bar{u}^{\prime }(t)-\bar{u}^{\prime }(s)|<C_\sigma |t-s|^{\frac{\sigma }{2}}$ for all $s,t\ge 1.$ We claim $\lim \limits _{t\rightarrow \infty }\bar{u}^{\prime }(t)=0$. Otherwise, there exists $\varepsilon >0$ and a sequence $t_k\rightarrow \infty$ ($t_k\ge 1$) such that $|\bar{u}^{\prime }(t_k)|>2\varepsilon$ for all $k\in \mathbb N_+$. Clearly, for any $t\ge 1$ satisfying $|t-t_k|<(\varepsilon /{C_\sigma })^{\frac{2}{\sigma }}=:\delta$ (note that $\delta$ is independent of *k*), we have

$$\begin{aligned} &  |\bar{u}^{\prime }(t)-\bar{u}^{\prime }(t_k)|<\varepsilon \quad \text {and} \\ &  \left| \bar{u}^{\prime }(t)\right| \ge \left| \bar{u}^{\prime }\left( t_k\right) \right| -\left| \bar{u}^{\prime }(t)-\bar{u}^{\prime }\left( t_k\right) \right| >\varepsilon . \end{aligned}$$

This along with $\bar{u}^{\prime }(\cdot )\in C^{\sigma / 2}([1, \infty ))$ indicates that $\bar{u}^{\prime }(t)$ will not change the sign in $[t_k,t_k+\delta ]$. Using (3.10), we obtain

$$\begin{aligned} \delta \varepsilon \le \int _{t_k}^{t_k+\delta }|\bar{u}^{\prime }(x)| d x= &  \left| \int _1^{t_k} \bar{u}^{\prime }(x) d x-\int _1^{t_k+\delta } \bar{u}^{\prime }(x) d x\right| \\= &  \left| \bar{u}\left( t_k\right) -\bar{u}\left( t_k+\delta \right) \right| \rightarrow 0\quad \text {as }k\rightarrow \infty , \end{aligned}$$

which is a contradiction. The claim $\lim \limits _{t\rightarrow \infty }\bar{u}^{\prime }(t)=0$ is proved. Hence (3.12) indicates that $\lim \limits _{t\rightarrow \infty }I_2(t)=0$, which gives

3.13 $$\begin{aligned} \lim \limits _{t\rightarrow \infty }\int _\Omega {wf_1(u)}=0. \end{aligned}$$

Moreover, the combination of (2.1), (3.10) and Lemma 3.4 implies that

3.14 $$\begin{aligned} \left| \int _\Omega w \left[ {f_1(u)-f_1(K_1)}\right] \right| \le C\Vert u-K_1\Vert _{L^{1}(\Omega )}\rightarrow 0\quad \text {as }t \rightarrow \infty . \end{aligned}$$

Therefore, we have from (3.13) and (3.14) that

$$\begin{aligned} 0 =\lim _{t \rightarrow \infty } \int _\Omega {wf_1(u)} -\lim _{t \rightarrow \infty } \int _\Omega w \left[ {f_1(u)-f_1(K_1)}\right] ={f_1(K_1)}\lim _{t \rightarrow \infty }\int _\Omega w, \end{aligned}$$

namely $\lim \limits _{t\rightarrow \infty }\Vert w\Vert _{L^{1}(\Omega )}=0$. This combined with Lemma 3.4 yields (3.11) and completes the proof. $\square$

### Global stability for $\theta < L$

For $\theta \in (0, L)$, we consider the global stability of the positive constant steady states. We start with the Holling type I functional response (1.4) and give the global stability of $P_*$.

#### Lemma 3.7

(Global stability of $P_*$) Assume that the assumptions in Theorem 2.1 hold with $f_1(u)$ and $f_2(v)$ given by (1.4), and (*u*, *v*, *w*) is the solution of system (1.3). Let $d_*$ be given by (2.2), $\theta \in (\theta _0,L)$, and $P_*=\left( u_*,v_*,w_*\right)$ be the unique positive constant steady state of system (1.3). Then for any $\sigma \in (0,1)$, it holds that

$$\begin{aligned} \lim _{t \rightarrow \infty }\left( \Vert u-u_*\Vert _{C^{2+\sigma }(\bar{\Omega })}+\Vert v-v_*\Vert _{C^{2+\sigma }(\bar{\Omega })}+\Vert w-w_*\Vert _{C^{2+\sigma }(\bar{\Omega })}\right) =0 \end{aligned}$$

if $d_3\ge d_*$, where “=" holds in the case of $\Vert u_0\Vert _{L^{\infty }(\Omega )}\le K_1$ and $\Vert v_0\Vert _{L^{\infty }(\Omega )}\le K_2$.

#### Proof

Let $E_s=P_*=\left( u_*,v_*,w_*\right)$ in (3.4) and (3.5). Then (3.5) implies $\Gamma _i=\beta _i$, $i=1,2$. Using (1.3), (1.4), (3.4), (3.5), the fact that

$$\begin{aligned} \theta =\beta _1f_1(u_*)+\beta _2f_2(v_*)=\alpha _1\beta _1 u_*+\alpha _2\beta _2 v_*,\quad 1=\frac{u_*}{K_1}+\alpha _1w_*=\frac{v_*}{K_2}+\alpha _2w_*, \end{aligned}$$

and integrating by parts, we have

3.15 $$\begin{aligned} {\mathcal {E}}' (t;P_*) =&~ \underbrace{-\beta _1d_1u_*\int _\Omega \frac{|\nabla u|^2}{u^2}-\beta _2d_2v_*\int _\Omega \frac{|\nabla v|^2}{v^2}-d_3w_*\int _\Omega \frac{|\nabla w|^2}{w^2}+w_*\int _\Omega \left( \chi _{1} \nabla u+\chi _{2} \nabla v\right) \cdot \frac{\nabla w}{w}}_{=:-\int _{\Omega } \aleph _1 \Xi _1 \aleph _1 ^{T}}\nonumber \\&+ \beta _1\int _\Omega \left( 1-\frac{u}{K_1}-\alpha _1 w\right) \left( u-u_*\right) +\beta _2\int _\Omega \left( 1-\frac{v}{K_2}-\alpha _2 w\right) \left( v-v_*\right) \nonumber \\&+\int _\Omega (\alpha _1\beta _1 u+\alpha _2\beta _2 v-\theta )(w-w_*) \nonumber \\ =&~ -\int _{\Omega } \aleph _1 \Xi _1 \aleph _1 ^{T}+ \beta _1\int _\Omega \left( 1-\frac{u}{K_1}-\alpha _1w_*\right) \left( u-u_*\right) +\beta _2\int _\Omega \left( 1-\frac{v}{K_2}-\alpha _2w_*\right) \left( v-v_*\right) \nonumber \\ =&~ -\int _{\Omega } \aleph _1 \Xi _1 \aleph _1 ^{T}-\frac{\beta _1}{K_1}\int _\Omega \left( u-u_*\right) ^2-\frac{\beta _2}{K_2}\int _\Omega \left( v-v_*\right) ^2\quad \text {for all }t>0, \end{aligned}$$

where $$\aleph _1:=\left( \nabla u,\nabla v,\nabla w\right) $$ and

$$\begin{aligned} \Xi _1:=\left( \begin{array}{ccc} \frac{\beta _1d_1u_*}{u^2} & 0& -\frac{\chi _1w_*}{2w}\\ 0 & \frac{\beta _2d_2v_*}{v^2} & -\frac{\chi _2w_*}{2w}\\ -\frac{\chi _1w_*}{2w} & -\frac{\chi _2w_*}{2w} & \frac{d_3w_*}{w^2} \end{array}\right) . \end{aligned}$$

By Sylvester’s criterion, the matrix $\Xi _1$ is non-negative definite if and only if the determinant of $\Xi _1$ satisfies

3.16 $$\begin{aligned} &  |\Xi _1|=\frac{\beta _1\beta _2d_1d_2u_*v_*w_*}{ u^2v^2w^2}\left[ d_3-\frac{w_*}{4}\left( \frac{\chi _1^2 u^2}{\beta _1 d_1u_*} +\frac{\chi _2^2 v^2}{\beta _2 d_2v_*}\right) \right] \ge 0. \end{aligned}$$

On one hand, if $\Vert u_0\Vert _{L^{\infty }(\Omega )}\le K_1$ and $\Vert v_0\Vert _{L^{\infty }(\Omega )}\le K_2$, then we have from (2.2), (3.2) and (3.16) that

$$\begin{aligned} |\Xi _1|\ge \frac{\beta _1\beta _2d_1d_2u_*v_*w_*}{ u^2v^2w^2}\left( d_3- d_* \right) \ge 0\quad \text {for all }(x, t) \in \bar{\Omega } \times (0,\infty ). \end{aligned}$$

On the other hand, if $\Vert u_0\Vert _{L^{\infty }(\Omega )}> K_1$ or $\Vert v_0\Vert _{L^{\infty }(\Omega )}> K_2$, then the comparison principle (e.g., see (Jin and Wang 2017, Lemma 2.2) for details) shows that

$$\begin{aligned} \limsup _{t\rightarrow \infty } u(x,t)\le K_1 \quad \text {and}\quad \limsup _{t\rightarrow \infty } v(x,t)\le K_2\quad \text {for all } x\in {\overline{\Omega }}. \end{aligned}$$

This along with (2.2), (3.2), (3.16) and $d_3>d_*$ indicates that there exists $T_1>0$ such that $|\Xi _1|\ge 0$ for all $(x, t) \in \bar{\Omega } \times [T_1,\infty )$. Therefore, $\Xi _1$ is non-negative definite, and hence (3.15) indicates that

$$\begin{aligned} {\mathcal {E}}' (t;P_*) \le -\frac{\beta _1}{K_1}\int _\Omega \left( u-u_*\right) ^2-\frac{\beta _2}{K_2}\int _\Omega \left( v-v_*\right) ^2\quad \text {for all }t\ge T_1. \end{aligned}$$

We are now in the same situation as (3.9), and the remaining proof is omitted here for brevity since it is similar to that of Lemma 3.6. $\square$

We next consider the Holling type II functional response (1.5), and give the global stability of $Q_*$.

#### Lemma 3.8

(Global stability of $Q_*$) Assume that the assumptions in Theorem 2.1 hold with $f_1(u)$ and $f_2(v)$ given by (1.5), and (*u*, *v*, *w*) is the solution of the system (1.3). Let $d_*$ be given by (2.2), $\theta \in (0,L)$, and $Q_*=\left( u_*,v_*,w_*\right)$ be a positive constant steady state of the system (1.3). Then for any $\sigma \in (0,1)$, the conclusion

$$\begin{aligned} \lim _{t \rightarrow \infty }\left( \Vert u-u_*\Vert _{C^{2+\sigma }(\bar{\Omega })}+\Vert v-v_*\Vert _{C^{2+\sigma }(\bar{\Omega })}+\Vert w-w_*\Vert _{C^{2+\sigma }(\bar{\Omega })}\right) =0 \end{aligned}$$

holds if $d_3\ge d_*$, where “=" holds in the case of $\Vert u_0\Vert _{L^{\infty }(\Omega )}\le K_1$ and $\Vert v_0\Vert _{L^{\infty }(\Omega )}\le K_2$.

#### Proof

Let $E_s=Q_*=\left( u_*,v_*,w_*\right)$ in (3.4) and (3.5). Then (3.5) implies $\Gamma _1=\frac{\beta _1}{1+\gamma _1h_1 u_*}$ and $\Gamma _2=\frac{\beta _2}{1+\gamma _2h_2 v_*}$. Using (1.3), (1.5), (3.4), (3.5), the fact that $\theta =\beta _1f_1(u_*)+\beta _2f_2(v_*)$, and integration by parts, we obtain

3.17 $$\begin{aligned} {\mathcal {E}}' (t;Q_*) =&~ \underbrace{-\Gamma _1d_1u_*\int _\Omega \frac{|\nabla u|^2}{u^2}-\Gamma _2d_2v_*\int _\Omega \frac{|\nabla v|^2}{v^2}-d_3w_*\int _\Omega \frac{|\nabla w|^2}{w^2} +w_*\int _\Omega \left( \chi _{1} \nabla u+\chi _{2} \nabla v\right) \cdot \frac{\nabla w}{w}}_{=:-\int _{\Omega } \aleph _2 \Xi _2 \aleph _2 ^{T}}\nonumber \\&\quad +\Gamma _1\int _\Omega \left( 1-\frac{u}{K_1}-\frac{wf_1(u)}{u}\right) \left( u-u_*\right) +\Gamma _2\int _\Omega \left( 1-\frac{v}{K_2}-\frac{wf_2(v)}{v}\right) \left( v-v_*\right) \nonumber \\&\quad + \beta _1\int _\Omega \left( f_1(u)-f_1(u_*)\right) \left( w-w_*\right) +\beta _2\int _\Omega \left( f_2(v)-f_2(v_*)\right) \left( w-w_*\right) . \end{aligned}$$

Similar to (3.8), it holds that

3.18 $$\begin{aligned} \beta _1\left( f_1(u)-f_1(u_*)\right)&=\Gamma _1\frac{f_1(u)}{u}(u-u_*) \quad \text {and}\quad \nonumber \\ \beta _2\left( f_2(v)-f_2(v_*)\right)&=\Gamma _2\frac{f_2(v)}{v}(v-v_*). \end{aligned}$$

Substituting (3.18) into (3.17), and using (2.3) and the fact that $w_*=\frac{u_*}{f_1(u_*)}(1-\frac{u_*}{K_1})=\frac{v_*}{f_2(v_*)}(1-\frac{v_*}{K_2})$, we obtain

$$\begin{aligned} {\mathcal {E}}' (t;Q_*)=&~ -\int _{\Omega } \aleph _2 \Xi _2 \aleph _2 ^{T} +\Gamma _1 \int _\Omega \left( 1-\frac{u}{K_1}-\frac{w_*f_1(u)}{u}\right) \left( u-u_*\right) \\&+\Gamma _2 \int _\Omega \left( 1-\frac{v}{K_2}-\frac{w_*f_2(v)}{v}\right) \left( v-v_*\right) \\ =&~ -\int _{\Omega } \aleph _2 \Xi _2 \aleph _2 ^{T} -\int _\Omega \frac{\Gamma _1h_1\gamma _ 1(\lambda _1+u_*-K_1+u)}{K_1(1+h_1\gamma _ 1u)}(u-u_*)^2\\&-\int _\Omega \frac{\Gamma _2h_2\gamma _ 2(\lambda _2+v_*-K_2+v)}{K_2(1+h_2\gamma _2v)}(v-v_*)^2\\ \le&~ -\int _{\Omega } \aleph _2 \Xi _2 \aleph _2 ^{T}-\frac{\Gamma _1h_1}{K_1}f_1(u)(u-u_*)^2- \frac{\Gamma _2h_2}{K_2}f_2(v)(v-v_*)^2, \end{aligned}$$

where $$\aleph _2:=\left( \nabla u,\nabla v,\nabla w\right) $$ and

$$\begin{aligned} \Xi _2:=\left( \begin{array}{ccc} \frac{\Gamma _1d_1u_*}{u^2} & 0& -\frac{\chi _1w_*}{2w}\\ 0 & \frac{\Gamma _2d_2v_*}{v^2} & -\frac{\chi _2w_*}{2w}\\ -\frac{\chi _1w_*}{2w} & -\frac{\chi _2w_*}{2w} & \frac{d_3w_*}{w^2} \end{array}\right) . \end{aligned}$$

Then

$$\begin{aligned} |\Xi _2| =&~\frac{\Gamma _1\Gamma _2d_1d_2u_*v_*w_*}{ u^2v^2w^2}\left[ d_3-\frac{w_*}{4}\left( \frac{\chi _1^2 u^2}{\Gamma _1 d_1u_*}+\frac{\chi _2^2 v^2}{\Gamma _2 d_2v_*}\right) \right] \nonumber \\ =&~\frac{\Gamma _1\Gamma _2d_1d_2u_*v_*w_*}{ u^2v^2w^2}\left[ d_3-\frac{w_*}{4}\left( \frac{\chi _1^2 u^2(1+\gamma _1h_1u_*)}{\beta _1 d_1u_*}+\frac{\chi _2^2 v^2(1+\gamma _2h_2v_*)}{\beta _2 d_2v_*}\right) \right] . \end{aligned}$$

The remaining proof can be completed by the arguments similar to those in the proof of Lemma 3.7, which we omit here for brevity. $\square$

#### Proofs of Theorem 2.2 and Theorem 2.3

The combination of Lemma 3.6 and Lemma 3.7 proves Theorem 2.2. Moreover, Theorem 2.3 is a consequence of Lemma 3.6 and Lemma 3.8. $\square$

## Linear instability analysis

This section is dedicated to investigating possible Turing instability of the system (1.3) with (1.4) or (1.5) induced by spatial dispersal including diffusion and prey-taxis to explore the biological implications underlying the spatial patterns. Turing instability arises from a constant steady state that remains stable for the non-spatial system but destabilizes under spatial perturbations. For the one predator-one prey spatial system with the Holling type I (i.e. Lotka-Volterra) functional response and no flux boundary conditions, it is well-known that there is no spatially inhomogeneous pattern (De Mottoni and Rothe 1979; Du and Shi 2006), which remains unchanged in the presence of prey-taxis (Jin and Wang 2017, 2021). Moreover, there is no Turing instability either if the functional response is of Holling type II, see Appendix A for detailed analysis. For the predator-mediated apparent competition model (1.3) with one predator and two prey, a natural question is whether the system (1.3) can induce the Turing instability. In other words, can the one predator-prey system be destabilized by the invasion of another prey species? We shall attempt this question by starting with linear stability analysis.

### Stability/instability of the non-spatial (ODE) system

We begin with the local/global stability of equilibria of the non-spatial system (1.2) with (1.4) or (1.5). For biological interest, the initial biomass of each species is assumed to be positive, namely $u(0),v(0), w(0)\ge 0 \ (\not \equiv 0)$. We first recall the following stability results for the equilibria $E_0$, $E_u$, $E_v$ and $E_{uv}$ (cf. Lou et al. 2025)

#### Proposition 4.1

For the non-spatial system (1.2) with either (1.4) or (1.5), the equilibria $E_0$, $E_u$ and $E_v$ are saddles, $E_{uv}$ is a saddle if $\theta <L$, and $E_{uv}$ is globally asymptotically stable if $\theta \ge L$.

#### Non-spatial system with the Holling type I functional response

Let $f_1(u)$ and $f_2(v)$ be given by (1.4). Then the global stability of solutions to (1.2) can be classified completely, as shown in Table 2.

**Table 2:** The stability of equilibria of the ODE system (1.2) with (1.4)

	$\theta \in (0,\theta _0]$	$\theta \in (\theta _0,L)$	$\theta \in [L,\infty )$
$\alpha _1>\alpha _2$	$P_2$ is GAS	2$P_*$is GAS	$E_{uv}$ is GAS
$\alpha _1<\alpha _2$	$P_1$ is GAS	$P_*$ is GAS	$E_{uv}$ is GAS
$\alpha _1=\alpha _2\ (\Longleftrightarrow \theta _0=0)$		$P_*$ is GAS	$E_{uv}$ is GAS

Note: The abbreviation “GAS” stands for “globally asymptotically stable”. The notation “$\Longleftrightarrow$” denotes “if and only if”

#### Non-spatial system with the Holling type II functional response

Unlike the Holling type I, the global dynamics of (1.2) with the Holloing type II functional response (1.5) can only be partially confirmed as shown in Table 3 for $E_{uv}$, $Q_1$, $Q_2$ and $Q_*$ (cf. Lou et al. 2025).

**Table 3:** The stability of equilibria of the ODE system (1.2) with (1.5)

$i\in \left\{ 1,2\right\}$	$\theta \in (0,L_i)$	$\theta \in [L_i,L)$	$\theta \in [L,\infty )$
$(K_1,K_2)\in \Lambda _i$	$Q_i$ is GAS	Unclear	$E_{uv}$ is GAS
$(K_1,K_2)\in \Lambda _*$	$Q_*$ is GAS	$Q_*$ is GAS	$E_{uv}$ is GAS
$(K_1,K_2)\not \in \Lambda _{1}\cup \Lambda _{2}\cup \Lambda _*$	Unclear	Unclear	$E_{uv}$ is GAS

Note: Here, $\Lambda _{1}:=\{(K_1,K_2)\mid K_1\le \lambda _1+u_{Q_1}, \frac{K_2}{f_2(K_2)}\le w_{Q_1}\}$, $\Lambda _{2}:=\{(K_1,K_2)\mid K_2\le \lambda _2+v_{Q_2}, \frac{K_1}{f_1(K_1)}\le w_{Q_2}\}$, and $\Lambda _*$ is defined by (2.3). The abbreviation “GAS” stands for “globally asymptotically stable”

As observed in Table 3, there are some parameter gaps where the global dynamics of the system (1.2) with (1.5) remain ambiguous. Indeed it is too complicated to derive affirmative results in these gaps for general parameters. Mediated by a shared predator species, apparent competition between two prey species may be symmetric or asymmetric (see (Holt and Bonsall 2017, Figure 1)), where symmetric (resp. asymmetric) apparent competition occurs when two prey species have the same (resp. different) ecological characteristics. For clarity and definiteness, the stability analysis was conducted in Lou et al. (2025) to explore the effect of the predator-mediated apparent competition on population dynamics in two specific parameter configurations characterizing symmetric and asymmetric apparent competition. Below we shall briefly recall these results to investigate the pattern formation driven by the dispersal strategies of species later.

- **Symmetric apparent competition.** With the parameter configuration
  4.1 $$\begin{aligned} K_1=K_2=3\quad \text {and}\quad \beta _1=\beta _2=h_1=h_2=\gamma _1=\gamma _2=1, \end{aligned}$$
  it follows that $L_1=L_2=\frac{3}{4}$ and $L=\frac{3}{2}$. The system (1.2) with (1.5) and (4.1) has the equilibria
  4.2 $$\begin{aligned} {\left\{ \begin{array}{ll} E_0, E_u, E_v, E_{uv}, Q_1, Q_2, {Q_*^0}, & \quad \text {if }\theta \in (0,\frac{2}{3}],\\ E_0, E_u, E_v, E_{uv}, Q_1, Q_2, {Q_*^0}, {Q_*^1}, {Q_*^2}, & \quad \text {if }\theta \in (\frac{2}{3},\frac{3}{4}),\\ E_0, E_u, E_v, E_{uv}, {Q_*^0}, {Q_*^1}, {Q_*^2}, & \quad \text {if }\theta \in [\frac{3}{4},1),\\ E_0, E_u, E_v, E_{uv}, {Q_*^0}, & \quad \text {if }\theta \in [1,\frac{3}{2}),\\ E_0, E_u, E_v, E_{uv}, & \quad \text {if }\theta \in [\frac{3}{2},\infty ),\\ \end{array}\right. } \end{aligned}$$
  where $$E_0=(0,0,0)$$, $$E_u=(3,0,0)$$, $$E_v=(0,3,0)$$, $$E_{uv}=(3,3,0)$$, and
  4.3 $$\begin{aligned} {\left\{ \begin{array}{ll} Q_1=\left( \frac{ \theta }{1-\theta },0,\frac{3-4\theta }{3 (1-\theta )^2}\right) ,\quad Q_2=\left( 0,\frac{ \theta }{1-\theta },\frac{3-4\theta }{3 (1-\theta )^2}\right) ,\\ {Q_*^0}=\left( \frac{\theta }{2-\theta },\frac{\theta }{2-\theta },\frac{4 (3-2 \theta )}{3 (2-\theta )^2}\right) ,\\ {Q_*^1}=\left( 1+2 \sqrt{\frac{1-\theta }{2-\theta }},1-2 \sqrt{\frac{1-\theta }{2-\theta }},\frac{4}{3(2-\theta )}\right) ,\ {Q_*^2}=\left( 1-2 \sqrt{\frac{1-\theta }{2-\theta }},1+2 \sqrt{\frac{1-\theta }{2-\theta }},\frac{4}{3(2-\theta )}\right) . \end{array}\right. } \end{aligned}$$
  The stability results of the above equilibria are summarized in Table 4.

**Table 4:** Stability of the equilibria of the ODE system (1.2) with (1.5) and (4.1)

Equilibria	$\theta$										
	$(0,\frac{1}{2})$	$\frac{1}{2}$	$(\frac{1}{2},\frac{2}{3})$	$\frac{2}{3}$	$(\frac{2}{3},\theta _1)$	$[\theta _1,\frac{3}{4})$	$[\frac{3}{4},1)$	1	$(1,\frac{4}{3})$	$[\frac{4}{3},\frac{3}{2})$	$[\frac{3}{2},\infty )$
$$E_0, E_u, E_v $$	Saddle	Saddle	Saddle	Saddle	Saddle	Saddle	Saddle	Saddle	Saddle	Saddle	Saddle
$E_{uv}$	Saddle	Saddle	Saddle	Saddle	Saddle	Saddle	Saddle	Saddle	Saddle	Saddle	GAS
$Q_1$, $Q_2$	SF	MS	S-FN	MS	SF	Saddle	/	/	/	/	/
${Q_*^0}$	U-FN	U-FN	U-FN	U-FN	U-FN	U-FN	U-FN	MS	S-FN	GAS	/
${Q_*^1},{Q_*^2}$	/	/	/	/	S-FN	S-FN	S-FN	/	/	/	/

Note: The abbreviations “SF", “MS", “S-FN", “U-FN" and “GAS" stand for “saddle-focus", “marginally stable", “stable focus node", “unstable focus node" and “globally asymptotically stable", respectively. The notation “/" denotes “equilibria do not exist”

- **Asymmetric apparent competition.** In this case, the parameter configuration is taken as
  4.4 $$\begin{aligned} K_1=K_2=h_1=h_2=1,\ \beta _1=\beta _2=b>0 \quad \text {and}\quad 0< \gamma _2< \gamma _1=1. \end{aligned}$$

Then $L_1=\frac{b}{2}> L_2=\frac{b \gamma _2}{1+\gamma _2},\ L= \frac{b (1+3 r_2)}{2 (1+r_2)}$, and system (1.2) with (1.5) and (4.4) has the equilibria

$$\begin{aligned} {\left\{ \begin{array}{ll} E_0, E_u, E_v, E_{uv}, Q_1, Q_2, & \quad \text {if }\theta \in (0,\theta _*],\\ E_0, E_u, E_v, E_{uv}, Q_1, Q_2, Q_*, & \quad \text {if }\theta \in (\theta _*,L_2),\\ E_0, E_u, E_v, E_{uv}, Q_1, Q_*, & \quad \text {if }\theta \in [L_2,L_1),\\ E_0, E_u, E_v, E_{uv}, Q_*, & \quad \text {if }\theta \in [L_1,L),\\ E_0, E_u, E_v, E_{uv}, & \quad \text {if }\theta \in [L,\infty ),\\ \end{array}\right. } \end{aligned}$$

where $$E_0=(0,0,0)$$, $$E_u=(1,0,0)$$, $$E_v=(0,1,0)$$, $$E_{uv}=(1,1,0)$$,

$$\begin{aligned} &  Q_1=\left( \frac{\theta }{b-\theta },0,\frac{b (b-2 \theta )}{(b-\theta )^2}\right) , \\ &  Q_2=\left( 0,\frac{\theta }{\gamma _2(b-\theta )},\frac{b (b \gamma _2-(1+\gamma _2) \theta )}{\gamma _2^2(b-\theta )^2}\right) , \end{aligned}$$

and

$$\begin{aligned}&\theta _* =\varphi _1(\gamma _2) b\in (0, L_2),\\&\varphi _1(\gamma _2):=\frac{ \sqrt{(1-\gamma _2) (3 \gamma _2+1)}-(1-\gamma _2) (2 \gamma _2+1)}{2 \gamma _2^2}\in (0,\frac{1}{4}]. \end{aligned}$$

The (unique) coexistence equilibrium $Q_*$ exists if and only if $\theta \in (\theta _*,L)$ (cf. (Lou et al. 2025, Appendix B)). In this case, the global population dynamics can be completely understood (see Lou et al. 2025), as shown in Table 5.

**Table 5:** Stability of equilibria of the ODE system (1.2) with (1.5) and (4.4)

$\theta$	$(0,\theta _*]$	$(\theta _*,L)$	$[L,\infty )$
Global stability	$Q_2$ is GAS	$Q_*$ is GAS	$E_{uv}$ is GAS

Note: The abbreviation “GAS" stands for “globally asymptotically stable"

### Stability and instability of the spatial (PDE) system

We next consider the system (1.3) with (1.4) or (1.5). When the spatial dimension $n\le 2$, Theorem 2.2(ii), Theorem 2.3(ii) and Remark 2.1 imply that for $\theta \ge L$, $E_{uv}$ remains globally asymptotically stable under spatial perturbations. This along with Proposition 4.1 shows that possible Turing instability can only arise from the constant semi-coexistence/coexistence steady states. We begin with the following general framework of linear stability analysis, where the spatial dimension $n\ge 1$ (note that the linear stability analysis does not require global boundedness).

#### General linear stability analysis

Linearize the system (1.3) at a constant steady state $E_s=(u_s,v_s,w_s)$ to get

4.5 $$\begin{aligned} \left\{ \begin{array}{llll} \Psi _{t}=\mathcal {M} \Delta \Psi +\mathcal {J} \Psi , & x \in \Omega , t>0, \\ {\partial _{\nu }} \Psi =0, & x \in \partial \Omega , t>0, \\ \Psi (x, 0)=\left( u_{0}-u_{s}, v_{0}-v_{s},w_{0}-w_{s}\right) ^{\mathcal {T}}, & x \in \Omega , \end{array} \right. \end{aligned}$$

where $\Psi (x, t)=\left( u-u_{s}, v-v_{s},w-w_{s}\right) ^{\mathcal {T}}$ and $\partial _{\nu }\Psi (x, t)=\left( \partial _{\nu } u, \partial _{\nu } v,\partial _{\nu } w\right) ^{\mathcal {T}}$ with $\mathcal {T}$ denoting the transpose, and the matrices $\mathcal {M}=\mathcal {M}(E_s)$ and $\mathcal {J}=\mathcal {J}(E_s)$ are given by

4.6 $$\begin{aligned} \mathcal {M}:= \left( \begin{array}{ccc} d_1 & 0& 0 \\ 0 & d_2& 0\\ -\chi _1w_{s}& -\chi _2w_{s}& d_3 \end{array} \right) ,\ \mathcal {J}&:= \left( \begin{array}{ccc} J_{11} & J_{12} & J_{13} \\ J_{21} & J_{22} & J_{23} \\ J_{31}& J_{32}& J_{33} \\ \end{array} \right) \end{aligned}$$

with

$$\begin{aligned} \left( \begin{array}{ccc} J_{11} & J_{12} & J_{13} \\ J_{21} & J_{22} & J_{23} \\ J_{31}& J_{32}& J_{33} \\ \end{array} \right) = \left( \begin{array}{ccc} 1-\frac{2 u_s}{K_1}-w_s f_1'(u_s) & 0 & -f_1(u_s) \\ 0 & 1-\frac{2 v_s}{K_2}-w_s f_2'(v_s) & -f_2(v_s) \\ \beta _1 w_s f_1'(u_s)& \beta _2 w_s f_2'(v_s) & \beta _{1} f_{1}(u_s)+\beta _{2} f_{2}(v_s)-\theta \\ \end{array} \right) . \end{aligned}$$

By the separation of variables, we know that the linear system (4.5) has the solution in the form of

$$\begin{aligned} \Psi (x, t)=\sum _{k \ge 0} \left( U_k,V_k,W_k\right) ^{\mathcal {T}} e^{{\rho _k} t} \zeta _k (x). \end{aligned}$$

Here ${\rho _k}$ is the temporal eigenvalue, $\left( U_k,V_k,W_k\right) ^{\mathcal {T}}$ are the coefficients of the Fourier expansion of the initial function $\Psi (x, 0)$ in terms of $\zeta _k (x)$ which are eigenfunctions of the eigenvalue problem

4.7 $$\begin{aligned} {\left\{ \begin{array}{ll} -\Delta \zeta _k (x)={\mu _k} \zeta _k (x), \quad & x\in \Omega ,\\ \partial _{\nu } \zeta _k (x) =0, \quad & x\in \partial \Omega , \end{array}\right. } \end{aligned}$$

where $\{\mu _k\}_{k\ge 0}$ denote the eigenvalues of $-\Delta$ under the Neumann boundary condition with $0=\mu _0<\mu _1 \le \mu _2 \le \mu _3 \le \ldots$. Substituting (4.7) into (4.5), we arrive at

$$\begin{aligned} {\rho _k} \zeta _k (x)=-{\mu _k} \mathcal {M} \zeta _k (x)+\mathcal {J} \zeta _k (x)=\mathcal {B} \zeta _k (x), \end{aligned}$$

with the matrix $\mathcal {B}$ given by

$$\begin{aligned} \mathcal {B}:=-{\mu _k} \mathcal {M}+\mathcal {J} = \left( \begin{array}{ccc} -d_1{\mu _k}+J_{11} & 0 & J_{13} \\ 0 & -d_2{\mu _k}+J_{22} & J_{23} \\ \chi _1w_s{\mu _k}+J_{31}& \chi _2w_s{\mu _k}+J_{32}& -d_3{\mu _k}+J_{33} \\ \end{array} \right) . \end{aligned}$$

Using (4.6) and denoting the three eigenvalue of $\mathcal {B}$ by ${\rho _k^0}$ and ${\rho _k^\pm }$, we know that ${\rho _k^0}$ and ${\rho _k^\pm }$ are the roots of

4.8 $$\begin{aligned} \begin{aligned} \varphi ({\rho _k}):={\rho _k^3}+A_2{\rho _k^2}+A_1{\rho _k}+A_0=0, \end{aligned} \end{aligned}$$

where

4.9 $$\begin{aligned} \left\{ \begin{array}{llll} A_2:=D_2 {\mu _k}-tr({\mathcal {J}}),\\ A_1:=D_1 {\mu _k^2}- \left( Y_1+X_1\right) {\mu _k}+Y_2,\\ A_0:= D_0 {\mu _k^3} - \left( Y_3+X_2\right) {\mu _k^2}+\left( Y_4+X_3\right) {\mu _k}-Det(\mathcal J), \end{array} \right. \end{aligned}$$

with

4.10 $$\begin{aligned} {\left\{ \begin{array}{ll} D_2=d_1+d_2+d_3>0,\quad D_1=d_1d_2+d_1d_3+d_2d_3>0,\quad D_0=d_1d_2d_3>0,\\ tr({\mathcal {J}}):=J_{11}+J_{22}+J_{33},\quad Det({\mathcal {J}}):=J_{11} J_{22} J_{33}-J_{11} J_{23} J_{32}-J_{13} J_{22} J_{31},\\ Y_1:=d_1\left( J_{22}+J_{33}\right) +d_2\left( J_{11}+J_{33}\right) +d_3\left( J_{11}+J_{22}\right) ,\\ Y_2:=J_{11} J_{22}+J_{11} J_{33}+J_{22} J_{33}-J_{13} J_{31}-J_{23} J_{32},\\ Y_3:=d_1d_2J_{33}+d_1d_3J_{22}+d_2d_3J_{11},\\ Y_4:=d_1\left( J_{22}J_{33}-J_{23}J_{32}\right) +d_2\left( J_{11}J_{33}-J_{13}J_{31}\right) +d_3J_{11}J_{22},\\ X_1:=\left( \chi _1 J_{13}+\chi _2 J_{23}\right) w_s,\quad X_2:=\left( d_2\chi _1 J_{13}+d_1\chi _2 J_{23}\right) w_s,\\ X_3:=\left( \chi _1 J_{13} J_{22}+\chi _2 J_{11} J_{23}\right) w_s. \end{array}\right. } \end{aligned}$$

It follows from the Routh-Hurwitz criterion (cf. (Murray 2002, Appendix B)) that all roots of (4.8) have negative real parts if and only if

4.11 $$\begin{aligned} A_2,A_1,A_0>0\quad \text {and}\quad A_*:= A_1A_2-A_0 =D_3 {\mu _k^3}+B_4 {\mu _k^2}+B_2 {\mu _k}+B_0>0, \nonumber \\ \end{aligned}$$

where

4.12 $$\begin{aligned} {\left\{ \begin{array}{ll} D_3:=D_1D_2-D_0=({d_1}+{d_2}) ({d_1}+{d_3}) ({d_2}+{d_3})>0,\\ B_4:=X_2+Y_3-D_2(X_1+Y_1)- tr({\mathcal {J}})D_1,\\ B_2:=tr({\mathcal {J}})(X_1+Y_1)+D_2Y_2-X_3-Y_4,\\ B_0:=Det({\mathcal {J}})-tr({\mathcal {J}})Y_2. \end{array}\right. } \end{aligned}$$

The following discussion is divided into two parts for Holling type I and II functional responses, respectively.

#### Spatial system with the Holling type I functional response

Let $f_1(u)$ and $f_2(v)$ be given by (1.4). In view of Table 2, we aim to explore whether there is Turing instability arising from $P_1$, $P_2$, and $P_*$ of the system (1.3). We first consider the linear stability of $P_1$ (resp. $P_2$) for $\theta \in (0,\theta _0]$ and $\alpha _1<\alpha _2$ (resp. $\alpha _1>\alpha _2$).

##### Lemma 4.2

Let $f_1(u)$ and $f_2(v)$ be given by (1.4), and let $d_i>0$ and $\chi _i \ge 0$. If $\theta \in (0,\theta _0]$ and $\alpha _1<\alpha _2$ (resp. $\alpha _1>\alpha _2$), then $P_1$ (resp. $P_2$) of system (1.3) is linearly stable.

##### Proof

We only prove for $P_1$, and the case of $P_2$ can be proved similarly. Let $E_s=P_1=\left( \frac{\theta }{\alpha _1\beta _1}, 0,\frac{L_1-\theta }{\alpha _1L_1}\right)$ in Sect. 4.2.1. Then we obtain that the eigenvalues of the matrix ${\mathcal {B}}$ are

$$\begin{aligned} {\rho _k^0}=-d_2 {\mu _k}+\frac{\alpha _2(\theta -\theta _0)}{\alpha _1L_1} \quad \text {and}\quad {\rho _k^\pm } =-\frac{1}{2L_1}\left[ \left( L_1(d_1+d_3){\mu _k}+\theta \right) \pm \sqrt{ I_3}\right] , \end{aligned}$$

where $I_3:= (d_1-d_3)^2L_1^2{\mu _k^2} +2\theta \left[ (d_1-d_3)L_1+2\chi _1K_1(\theta -L_1)\right] {\mu _k} +[4L_1(\theta -L_1) +\theta ]\theta$. For Turing instability, we only concern ${\mu _k}>0$. Clearly, ${\rho _k^0}\le -d_2 {\mu _k}<0$ for $\theta \in (0,\theta _0]$. Moreover, if $I_3\le 0$, then $\textrm{Re}({\rho _k^\pm })<0$; if $I_3>0$, it also holds that $\textrm{Re}({\rho _k^\pm })<0$ since $0<\theta \le \theta _0=\left( 1-\frac{\alpha _1}{\alpha _2}\right) L_1<L_1$ and

$$\begin{aligned} \left( L_1(d_1+d_3){\mu _k}+\theta \right) ^2-I_3 =&~ 4d_1d_3L_1^2{\mu _k^2}+4\theta \left[ d_3L_1+\chi _1K_1\left( L_1-\theta \right) \right] {\mu _k}\\ &+4L_1\theta \left( L_1-\theta \right) \nonumber \\ \ge&~ 4L_1\theta \left( L_1-\theta \right) >0. \end{aligned}$$

This completes the proof. $\square$

We then consider the linear stability of $P_*$ for $\theta \in (\theta _0, L)$.

##### Lemma 4.3

Let $f_1(u)$ and $f_2(v)$ be given by (1.4), and let $d_i>0$ and $\chi _i \ge 0$. If $\theta \in (\theta _0, L)$, then the unique positive constant steady state $P_*$ of the system (1.3) is linearly stable.

##### Proof

Let $E_s=P_*=(u_*,v_*,w_*)$ in Sect. 4.2.1. Then (4.6) implies

4.13 $$\begin{aligned} \mathcal {J} = \left( \begin{array}{ccc} -\frac{u_*}{K_1} & 0 & -\alpha _1 u_* \\ 0 & -\frac{v_*}{K_2} & -\alpha _2 v_* \\ \alpha _1\beta _1w_*& \alpha _2\beta _2w_*& 0 \\ \end{array} \right) . \end{aligned}$$

Therefore, we have from (4.10) that

$$\begin{aligned} -tr(\mathcal {J}),\ -(Y_1+X_1),\ Y_2,\ -(Y_3+X_2),\ (Y_4+X_3),\ -Det(\mathcal {J}) >0, \end{aligned}$$

which alongside (4.9) implies $A_2,A_1,A_0>0$. For $A_*=A_1 A_2-A_0=D_3 {\mu _k^3}+B_4 {\mu _k^2}+B_2 {\mu _k}+B_0$ given by (4.11), we have from (4.10), (4.12) and (4.13) that

$$\begin{aligned} B_4&> X_2+Y_3-D_2(X_1+Y_1) \\&= \frac{( D_1+d_2^2 + d_3^2) u_*}{K_1}+\frac{( D_1+ d_1^2+d_3^2) v_*}{K_2} \nonumber \\ &\quad +\alpha _1 ( d_1+ d_3)u_* w_* \chi _1+\alpha _2 ( d_2+ d_3) v_* w_* \chi _2>0,\nonumber \\ B_2&= \frac{(d_1 +d_2)u_* v_*+\alpha _1 K_2 u_*^2 w_* \chi _1+\alpha _2 K_1 v_*^2 w_* \chi _2}{K_1K_2} \nonumber \\ &\quad +\left[ \alpha _1 ^2 \beta _1 u_* ( d_1+ d_3)+\alpha _2 ^2 \beta _2 v_* ( d_2+ d_3)\right] w_*>0,\nonumber \\ B_0&=\frac{w_* \left( \alpha _{1}^2 \beta _{1} K_{2} u_*^2+\alpha _{2}^2 \beta _{2} K_{1} v_*^2\right) }{ K_{1} K_{2}}+\frac{u_* v_* ( K_{1} v_*+ K_{2} u_*)}{ K_{1}^2 K_{2}^2}>0. \end{aligned}$$

Therefore, $A_*=A_1 A_2-A_0>0$. Now (4.11) is satisfied and the proof is completed. $\square$

##### Remark 4.1

The above analysis indicates that the system (1.3) with the Holling type I functional response (1.4) has no Turing instability.

#### Spatial system with the Holling type II functional response

Let $f_1(u)$ and $f_2(v)$ be given by (1.5). Then we investigate whether system (1.3) admits Turing instability arising from $Q_1$, $Q_2$, and $Q_*$ for the symmetric parameter configuration (4.1) and the asymmetric one (4.4) with general spatial dispersal coefficients

4.14 $$\begin{aligned} d_1,d_2,d_3>0\quad \text {and}\quad \chi _1,\chi _2\ge 0. \end{aligned}$$

Below we shall focus on the symmetric parameter configuration (4.1), and give some brief remarks for the asymmetric one (4.4). Given the results in Table 4, we need to examine the linear stability of the following constant steady states: $Q_1$ and $Q_2$ for $\theta \in [\frac{1}{2},\frac{2}{3}]$; ${Q_*^0}$ for $\theta \in [1,\frac{3}{2})$; ${Q_*^1}$ and ${Q_*^2}$ for $\theta \in (\frac{2}{3},1)$.

##### Lemma 4.4

Let $f_1(u)$ and $f_2(v)$ be given by (1.5), and let (4.1) and (4.14) hold. Then the following results hold.

(i) The semi-coexistence constant steady states $Q_1$ and $Q_2$ of the system (1.3) are marginally stable for $\theta =\frac{1}{2},\frac{2}{3}$, and linearly stable for $\theta \in \left( \frac{1}{2},\frac{2}{3}\right)$.
(ii) The positive constant steady state ${Q_*^0}$ is marginally stable for $\theta =1$, and linearly stable for $\theta \in \left( 1,\frac{3}{2}\right)$.

##### Proof

The proof is similar to that of Lemmas 4.2 and 4.3 and is omitted for brevity. $\square$

Lemma 4.4 show that there is no Turing instability arising from $Q_1$, $Q_2$ and ${Q_*^0}$. We shall see below that Turing instability may arise from ${Q_*^1}$ and ${Q_*^2}$. Since the two positive constant steady states ${Q_*^1}$ and ${Q_*^2}$ are symmetric in the first two components and the third component is the same, we only conduct stability analysis for ${Q_*^1}$, and the case for ${Q_*^2}$ is similar.

**Instability arising from** ${Q_*^1}$. In view of (4.2), ${Q_*^1}$ exists if and only if $\theta \in (\frac{2}{3},1)$. To avoid excessive technicalities, we let $\theta =\frac{7}{8}\in (\frac{2}{3},1)$. Then with the symmetric parameters in (4.1), we consider

4.15 $$\begin{aligned} \theta =\frac{7}{8},\ {d_1,d_2,d_3>0},\ \chi _i\ge 0,\ K_i=3,\ \beta _i=h_i=\gamma _i=1,\quad i=1,2, \qquad \end{aligned}$$

which implies

$$\begin{aligned} {Q_*^1}=\left( \frac{5}{3},\frac{1}{3},\frac{32}{27}\right) . \end{aligned}$$

Let $E_s={Q_*^1}$ in Sect. 4.2.1. Using (4.9), (4.11) and (4.15), we have

4.16 $$\begin{aligned} {\left\{ \begin{array}{ll} A_2=D_2 {\mu _k}+\frac{1}{6}>0,\\ A_1=D_1 {\mu _k^2} +\left( \frac{-2d_1+5d_2+3d_3}{18}+\frac{20 \chi _1+8 \chi _2}{27}\right) {\mu _k} +\frac{311}{1296},\\ A_0=D_0 {\mu _k^3} +\left( \frac{(-2d_1+5d_2) {d_3}}{18}+\frac{20 d_2 \chi _1+8 d_1 \chi _2}{27} \right) {\mu _k^2} \\ \qquad \quad + \left( \frac{{d_1}}{6} +\frac{5 {d_2}}{48}-\frac{5 {d_3}}{162}+\frac{20 ( - \chi _1+\chi _2)}{243} \right) {\mu _k} +\frac{5}{144},\\ A_*=D_3 {\mu _k^3} +\left( \frac{-2d_1^2+5d_2^2+3d_3^2+6D_1}{18}+\frac{20 ( d_1+ d_3)\chi _1+8 ( d_2+ d_3)\chi _2}{27} \right) {\mu _k^2}\\ \qquad \ \ +\left( \frac{71 {d_1}+236 {d_2}+387 {d_3}}{1296}+\frac{50 \chi _1-8 \chi _2}{243} \right) {\mu _k} +\frac{41}{7776}. \end{array}\right. } \end{aligned}$$

Then we have the following result from (4.11) immediately.

##### Lemma 4.5

Let $f_1(u)$ and $f_2(v)$ be given by (1.5). With the parameter configuration (4.15), the following stability results hold for the positive constant steady state ${Q_*^1}$ of (1.3).

(i) ${Q_*^1}$ is linearly stable if and only if $\min \limits _{{\mu _k}>0}\left\{ A_0,{A_1,}A_*\right\} >0$.
(ii) Turing instability will arise from ${Q_*^1}$ if and only if
  4.17 $$\begin{aligned} \min \limits _{{\mu _k}>0}\left\{ A_0,{A_1,}A_*\right\} <0. \end{aligned}$$

##### Remark 4.2

It can be seen from (4.16) that there are many values of $(d,d_3,\chi _1,\chi _2)\in (0,\infty )^2\times [0,\infty )^2$ such that (4.17) holds. Moreover, any of $\min \limits _{{\mu _k}>0}A_0$, $\min \limits _{{\mu _k}>0}A_1$ and $\min \limits _{{\mu _k}>0}A_*$ may be negative. Moreover, Turing patterns can be driven by pure diffusion (i.e., $\chi _1=\chi _2=0$), which will be discussed in detail in Section 5.2.1.

##### Remark 4.3

The system (1.3) with the Holling type II functional response (1.5) and the symmetric parameter values in (4.15) has no Turing instability arising from $Q_1$, $Q_2$ and ${Q_*^0}$, but may induce Turing instability from ${Q_*^1}$ and ${Q_*^2}$ by diffusion or prey-taxis (see Lemma 4.5 and Remark 4.2). For the asymmetric parameters given in (4.4), Table 5 shows that the system (1.3) will globally asymptotically stabilize to $Q_2$, $Q_*$ or $E_{uv}$ depending on the value of $\theta$. With spatial movements (diffusion and prey-taxis), stability analysis shows that $Q_2$, $Q_*$ or $E_{uv}$ are still linearly stable (omitted here for brevity) and hence no spatial patterns can bifurcate from $Q_2$, $Q_*$ or $E_{uv}$.

## Numerical simulations and spatiotemporal patterns

Remark 4.1 indicates that for the Holling type I functional response (1.4), the system (1.3) cannot generate Turing patterns. However, the pattern is possible with the Holling type II functional response (1.5) if the apparent competition is asymmetric (see Remark 4.3). In this section, we use numerical simulations to illustrate spatiotemporal patterns generated by the system (1.3) with the Holling type II functional response (1.5) and investigate the effects of spatial movement on the global population dynamics.

### Onset of predator-mediated apparent competition

From our previous work (Lou et al., 2025), it is demonstrated that the predator-mediated apparent competition in the absence of spatial components is not necessarily effective since the invasive prey species may not successfully invade. Hence the first question concerned is whether the spatial movement (diffusion and/or prey-taxis) can impact the success/failure of invasion. Therefore, we need to examine the stability of the semi-coexistence constant steady state $Q_1=(\frac{ \theta }{1-\theta },0,\frac{3-4\theta }{3 (1-\theta )^2})$ given by (4.3). Sect. 5 shows that for all $\theta >0$ and spatial coefficients $(d_1,d_2,d_3,\chi _1,\chi _2)\in (0,\infty )^3\times [0,\infty )^2$, $Q_1$ is linearly stable for the system (1.3) with (1.5). We shall perform numerical simulations to see the possible patterns. Without loss of generality, we let $\Omega =(0,30\pi )$ and $\theta =\frac{1}{4}$, which gives $Q_1=(\frac{1}{3},0,\frac{32}{27})$. For definiteness, we set $d_1=d_2=d_3=1$ and set the initial data as

5.1 $$\begin{aligned} (u_0,v_0,w_0)=Q_1+(0,R+0.01\cdot \cos (\pi x),0),\quad x\in \Omega , \end{aligned}$$

where $R>0$ represents the biomass of the invasive prey species to be chosen later. Now we study the effect of prey-taxis on predator-mediated apparent competition. We mainly discuss two cases: $(\chi _1,\chi _2)=(2,1)$ and $(\chi _1,\chi _2)=(10,1)$.

With $(\chi _1,\chi _2)=(2,1)$, which indicates that the strength of prey-taxis on the native prey species is “slightly" stronger than that on the invasive prey species, we observe the following two prominent phenomena from the numerical simulations shown in Fig. 1.

1. The initial biomass of invasive prey species plays a key role in determining the success/failure of invasions: small invasion biomass leads to failed invasions; medium and large invasion biomass leads to successful invasions and the native prey species are wiped out.
2. The spatially homogeneous time-periodic patterns always asymptotically appear regardless of whether the invasion is successful or not. This implies that the long-time population dynamics are mainly dominated by the corresponding ODE (non-spatial) system and the spatial effect is negligible in the long run.

For $(\chi _1,\chi _2)=(10,1)$, namely the strength of prey-taxis for the native prey species is much stronger than that for the invasive prey species, the numerical simulations are shown in Fig. 2, where the following phenomena can be found.

(i) The biomass of the invasive prey species is still a key factor determining whether the invasion is successful, same as in the former case $(\chi _1,\chi _2)=(2,1)$.
(ii) Prey-taxis can induce spatially inhomogeneous patterns, see Fig. 2**(**a).

### Instability driven by diffusion or prey-taxis

Based on the analyses in Sect. 5, we know that the system (1.3) with the Holling type I functional response (1.4) has no Turing instability. For the system (1.3) with the Holling type II functional response (1.5), Lemma 4.5(ii) shows that the instability of ${Q_*^1}$ may be driven by diffusion and/or prey-taxis in the case of symmetric apparent competition with parameter configuration (4.15). Next we shall show that the sole diffusion-drive instability is possible without prey-taxis (i.e. $\chi _1=\chi _2=0$), and further investigate the influence of prey-taxis on the pattern formation.

#### Diffusion-driven instability

With Lemma 4.5 and (4.16), there are many values of $d_1,d_2,d_3>0$ with $\chi _1=\chi _2=0$ which can satisfy (4.17) and hence give rise to Turing instability. We particularly consider three typical sets of diffusion rates as follows:

5.2 $$\begin{aligned} {\left\{ \begin{array}{ll} {\textbf {E.1}}: (d_1,d_2,d_3)=(1,1,60),\\ {\textbf {E.2}}: (d_1,d_2,d_3)=(60,1,1),\\ {\textbf {E.3}}: (d_1,d_2,d_3)=(60,\frac{1}{60},1). \end{array}\right. } \end{aligned}$$

It can be shown that system (1.3) with (1.5) and parameter setting given (4.15) and (5.2) may generate various bifurcations from ${Q_*^1}$, as summarized in Table 6.

**Table 6:** Turing bifurcations arising from system (1.3) with (1.5), (4.15) and (5.2), where $\chi _1=\chi _2=0$

$(d_1,d_2,d_3)$	**E.1**	**E.2**	**E.3**
$\min \limits _{{\mu _k}>0}A_1$	+	+	+
$\min \limits _{{\mu _k}>0}A_0$	−	$+$	−
$\min \limits _{{\mu _k}>0}A_*$	$+$	−	−
Bifurcation	Steady-state bifurcation	Hopf bifurcation	Steady-state bifurcation and Hopf bifurcation

##### Remark 5.1

It is known that the spatial one predator-one prey systems without prey-taxis do not have Turing instability (see Appendix A). However, in the system (1.3), where two prey species have negative indirect interactions mediated by one shared predator, diffusion can induce various Turing bifurcations resulting in profound spatial or periodic patterns as illustrated in Fig. 3.

We next consider the role of prey-taxis. It turns out that the interactions between prey-taxis and diffusion are quite delicate. For given diffusion coefficients, different values of $\chi _1$ and $\chi _2$ may have different effects (stabilization or destabilization) as demonstrated below.

#### Prey-taxis driven instability

We now show the prey-taxis may destabilize the system (1.3) with (1.5). We shall choose $\chi _1,\chi _2\ge 0$ as the bifurcation parameters to study the prey-taxis driven instability arising from $Q_*^1$. We assume the two prey species have the same diffusion rates, that is, $d_1=d_2=d>0$. For the sake of presentation, we denote

5.3 $$\begin{aligned} {\mathcal {S}}_1:=&~\left\{ k~\bigg |~ 0<{\mu _k}<\frac{1}{9d} \right\} ,\ {\mathcal {S}}_2:=\left\{ k~\bigg |~ 0<{\mu _k}<\frac{1}{9(d+d_3)} \right\} ,\nonumber \\ \chi _1^*=&~\chi _1^*(\chi _2,d,d_3):=\min _{k\in {\mathcal {S}}_1}\left\{ \frac{I_4}{320 {\mu _k} \left( 1-9 d {\mu _k}\right) }+\frac{ (18 d {\mu _k}+5)\chi _2}{5 \left( 1-9 d {\mu _k}\right) } \right\} , \nonumber \\ \chi _2^*=&~\chi _2^*(\chi _1,d,d_3):=\min _{k\in {\mathcal {S}}_2}\left\{ \frac{5 \chi _1 \left( 18 {\mu _k} (d+ d_3)+5\right) }{4 \left( 1-9 {\mu _k} (d+ d_3)\right) }+\frac{I_5}{256 {\mu _k} \left( 1-9 {\mu _k} (d+ d_3)\right) } \right\}>0,\nonumber \\ I_4:=&~-24 d_3 {\mu _k} \left( 1-9 d {\mu _k}\right) \left( 18 d {\mu _k}+5\right) +27 \left( 39 d {\mu _k}+5\right) ,\nonumber \\ I_5:=&~1296 {\mu _k^2} \left( 3 d^2+4 d d_3+ d_3^2\right) +15552 d {\mu _k^3} (d+ d_3)^2+6 {\mu _k} (307 d+387 d_3)+41>0. \end{aligned}$$

Then we can make the conclusions of Lemma 4.5(ii) more specific as follows.

##### Lemma 5.1

Let $f_1(u)$ and $f_2(v)$ be given by (1.5). For the parameter setting (4.15) with with $d_1=d_2=d>0$, the positive equilibrium ${Q_*^1}$ of the system (1.3) is linearly unstable if and only if

5.4 $$\begin{aligned} \text {either}\quad \min _{{\mu _k}>0}A_0<0 \quad \text {or}\quad \min _{{\mu _k}>0}A_*<0. \end{aligned}$$

Moreover, the following results hold.

- The steady-state bifurcation occurs (i.e. $\min \limits _{{\mu _k}>0}A_0<0$) if and only if
  $$\begin{aligned} {\mathcal {S}}_1\ne \emptyset \quad \text { and }\quad (\chi _1,\chi _2)\in \Omega _1:= \left\{ (\chi _1,\chi _2)\in [0,\infty )^2 \mid \chi _1>\chi _1^*(\chi _2,d,d_3)\right\} . \end{aligned}$$
- The Hopf bifurcation occurs (i.e. $\min \limits _{{\mu _k}>0}A_*<0$) if and only if
  $$\begin{aligned} {\mathcal {S}}_2\ne \emptyset \quad \text { and }\quad (\chi _1,\chi _2)\in \Omega _2:= \left\{ (\chi _1,\chi _2)\in [0,\infty )^2 \mid \chi _2>\chi _2^*(\chi _1,d,d_3)\right\} . \end{aligned}$$

##### Proof

Clearly, (4.16) with $d_1=d_2=d>0$ implies that $A_2,A_1>0$. Moreover, elementary analysis shows that

$$\begin{aligned} \max \left\{ \min _{{\mu _k}>0}A_0,\min _{{\mu _k}>0}A_*\right\}>0\quad \text {for all }d,d_3>0,\ \chi _1,\chi _2\ge 0. \end{aligned}$$

Therefore, Lemma 4.5 implies that ${Q_*^1}$ is linearly unstable if and only if (5.4) holds. The proofs for (a) and (b) are straightforward with tedious computations and we omit the details. $\square$

In the absence of prey-taxis (i.e. $\chi _1=\chi _2=0$), Lemma 5.1 indicates that the Hopf bifurcation can never occur due to $\chi _2^*>0$, and (5.3) implies that the steady-state bifurcation occurs if and only if

5.5 $$\begin{aligned} d_3>d_3^*:=\min _{k\in {\mathcal {S}}_1}\phi _2({\mu _k},d),\quad \phi _2({\mu _k},d):= \frac{9 \left( 39 d {\mu _k}+5\right) }{8 {\mu _k} \left( 1-9 d {\mu _k}\right) \left( 18 d {\mu _k}+5\right) }. \end{aligned}$$

It can be checked that $\frac{\partial \phi _2({\mu _k},d)}{\partial d}>0$ for $0<{\mu _k}<\frac{1}{9d}$. Without loss of generality, we let $d_1=d_2=d=1$. Then

$$\begin{aligned} d_3^* =\min _{0<{\mu _k}<\frac{1}{9}} \phi _2\left( {\mu _k},1\right) \approx \phi _2\left( {\mu _k},1\right) |_{{\mu _k}\approx 0.0517}\approx 48.1461. \end{aligned}$$

##### Remark 5.2

In an interval $\Omega =(0, \ell )$ with $\ell >0$, the following conclusions can be drawn from Lemma 5.1.

(i) If $\ell \in (0,3\pi ]$, there is no Turing instability.
(ii) If $\ell \in (3\pi ,3\sqrt{1+d_3}\pi ]$, only the steady-state bifurcation can occur.
(iii) If $\ell >3\sqrt{1+d_3}\pi$, either the steady-state bifurcation or Hopf bifurcation may occur.

We still take $l=30\pi$ so that two types of bifurcations may occur. Without prey-taxis ($\chi _1=\chi _2=0$), (5.5) gives

$$\begin{aligned} d_3^*=\min \limits _{0<{\mu _k}<\frac{1}{9}} \phi _2\left( {\mu _k},1\right) = \phi _2\left( \left( \frac{7\pi }{l}\right) ^2,1\right) \approx 48.2626. \end{aligned}$$

Consider two typical cases:

$$\begin{aligned} d_3=1<d_3^*,\quad d_3=60>d_3^*, \end{aligned}$$

where the former (resp. latter) implies $Q_*^1$ is linearly stable (resp. unstable) by Lemma 5.1. The numerical simulation for $d_3=60$ was already shown in Fig. 3(a). Then geometric illustration of the instability parameter regions $\Omega _1$ and $\Omega _2$ in the $\chi _1$-$\chi _2$ plane are shown in Fig. 4. Clearly, $\Omega _1\cap \Omega _2=\emptyset$ due to (5.4). Denoting

$$\begin{aligned} { l_i} := \left\{ (\chi _1,\chi _2)\in [0,\infty )^2 \mid \chi _i=\chi _i^*(\chi _j{,d,d_3})\right\} ,\ i+j=3,\ i=1,2, \end{aligned}$$

we can verify that the curve ${ l_2}$ lies above ${ l_1}$, as shown in Fig. 4. We denote the region bounded by ${ l_i} (i=1,2)$ and $\chi _i (i=1,2)$ axes by $\Omega _3$. Then $Q_1^*$ is linearly stable and there is no pattern formation in $\Omega _3$.

The above analyses indicate that prey-taxis can drive the instability of constant steady states of from system (1.3) with the Holling type II functional response (1.5) under certain parameter regions such as the parameter configuration given in (4.15) with $d_1=d_2=1$. These instabilities may result in the steady-state or Hopf bifurcations. Next we perform numerical simulations to illustrate the possible emerging spatial patterns. To this end, we set the parameter values as

5.6 $$\begin{aligned} \theta =\frac{7}{8},\ d_1=d_2=d_3=1, \ K_i=3,\ \beta _i=h_i=\gamma _i=1,\quad i=1,2. \end{aligned}$$

With parameters chosen in (5.6), we see that $$d_3=1<d_3^*$$ and hence diffusion can not induce Turing instability if $$\chi _1=\chi _2=0$$ (see the above discussions). Now we take the values of $$(\chi _1,\chi _2)$$ on the vertical line $$L\chi _1:\chi _1=30$$ in Fig. 4(b) and $$\chi _2=3,6,500$$, where $$(\chi _1=30,\chi _2=1)\in \Omega _1$$, $$(\chi _1=10,\chi _2=6)\in \Omega _3$$ and $$(\chi _1=30,\chi _2=500)\in \Omega _2$$. For $$\chi _2=3$$, the steady-state bifurcation occurs, and the corresponding numerical simulations are shown in Fig. 5(a), where non-constant stable spatial patterns are observed. When $$\chi _2$$ increases along the vertical line $$L\chi _1$$ in Fig. 4(b) and crosses the steady-state bifurcation boundary curve $${ l_1}$$ at the point $$O_1$$ into region $$\Omega _3$$, then the solution of (1.3) stabilizes to the positive constant steady state $${Q_*^1}$$. However, when $$\chi _2$$ further increases along the vertical line $$L\chi _1$$ and crosses the Hopf bifurcation boundary curve $${ l_2}$$ at the point $$O_2$$ into the region $$\Omega _2$$, the Hopf bifurcation will occur and patterns will arise from $${Q_*^1}$$ as shown in Fig. 5(c) where we observe the temporally oscillatory and spatially inhomogeneous patterns. Therefore, fixing $$\chi _1>0$$, we see that only when $$\chi _2$$ is small (i.e. below the curve $${ l_1}$$) or large (i.e. above the curve $$ l_2$$), the instability can occur and spatial patterns arise. This is similar to $$\chi _1>0$$ by fixing $$\chi _2>0$$, see Fig. 4(a)-(b). However if $$(\chi _1, \chi _2)\in \Omega _3$$ (i.e. the values of $$\chi _1$$ and $$\chi _2$$ are moderate), there is no spatial patterns.

#### Prey-taxis driven stability

We still consider the three examples in (5.2) with parameter configuration (4.15). For these parameter values, the analysis in Sect. 5.2.1 says that $Q_*^1$ with $\chi _1=\chi _2=0$ is linearly unstable, and hence spatial patterns will develop from $Q_*^1$ as shown in Fig. 3. Now we let $\chi _1, \chi _2>0$ and keep other parameter values the same. For definiteness, we fix $\chi _1=\chi _2=10$ without loss of generality. Then it can be checked that $\min \limits _{{\mu _k}>0}\left\{ A_0,{A_1,}A_*\right\} >0$ (calculations are routine and omitted here for brevity), and hence $Q_*^1$ is linearly stable by Lemma 4.5. This implies that the prey-taxis plays a stabilization role in this case. To make this visulizable, we consider a specific example **E.1** in (5.2) with parameter values (4.15) by choosing $d_1=d_2=1$ and $d_3=60$ and plot the instability/stability parameter regions in Fig. 4(c)-(d) where we see that the instability region $\Omega _1$ contains the origin $(\chi _1, \chi _2)=(0,0)$. If we increase the value of $\chi _2$ so that $(\chi _1, \chi _2)$ falls inside $\Omega _3$, then $Q_1^*$ becomes stable and spatial patterns may stabilize into the constant $Q_1^*$ as demonstrated by the numerical simulations shown in Fig. 5(b).

##### Remark 5.3

The above analyses and results along with numerical simulations show that prey-taxis in the predator-mediated apparent competition system may play complex roles. For instance, if the parameter values are given by (4.15), we find that prey-taxis may induce the instability if $(\chi _1,\chi _2)$ falls inside $\Omega _1$ or ${\Omega _2}$ while stability if $(\chi _1,\chi _2)\in \Omega _3$. This contrasts with one predator-one prey systems with prey-taxis which plays a single role, either stabilization (cf. Cai et al. 2022) or destabilization (cf. Song and Tang 2017). These observations along with the statement in Remark 5.1 assert that both diffusion and prey-taxis in one predator-two prey systems with the predator-mediated apparent competition can play significant roles different from the one predator-one prey systems. This implies that spatial movements will play more profound roles with the increasing number of species.

## Summary and discussion

In our previous work (Lou et al., 2025), we considered a temporal system (i.e. ODE counterpart of (1.3)) to explore the effects and biological consequences of the predator-mediated apparent competition (i.e. two prey species have an indirect negative interaction mediated by a shared predator species). The local and global stability of the equilibria of the temporal system with Holling type I and II functional responses were established and numerical simulations were performed to demonstrate the population dynamics and biological consequences caused by the predator-mediated apparent competition. However, the spatial movement of species, which is an indispensable factor in applications, was not considered in Lou et al. (2025). The goal of this paper is to include diffusion and prey-taxis into the ODE (temporal) system, leading to the system (1.3), and explore the spatial effects on the population dynamics and distributional structures.

With Holling type I and II functional responses, we establish the global stability of the coexistence (i.e. positive) and the predator-free constant steady states in certain parameter regimes. This yields a threshold dynamics in terms of the predator’s death rate, addressing under what conditions the spatial homogeneity can be achieved. Outside these parameter regimes, we conduct linear analysis to find the instability parameter regions by which we perform numerical simulations to demonstrate various intricate patterns arising from (1.3) with the Holling II functional response. With these numerical simulations, we find some interesting biological implications as summarized in (f.2)-(f.4) in Sect. 2.2. However, these implications are derived from numerical simulations or local instability analysis. Hence how to mathematically justify these patterns (like existence or stability) remains an interesting question. In particular, we find that spatial movements, including diffusion and prey-taxis, in the predator-prey system with two prey species will have significantly different effects from the system with one prey species. This implies that the effect of spatial movements can be distinct in complex ecological predator-prey systems depending on the number of species, and hence suggests that oversimplified models may not be adequate to describe the complex behavior of real ecosystems. More complicated models, though mathematically challenging, are often useful to gain a deeper understanding of the complex dynamics of realistic ecological systems.

## Data Availability

The authors declare that the manuscript has no associated data.

## Appendix A. Stability of one predator-one prey systems with prey-taxis

This appendix is devoted to investigating whether there is Turing instability arising from the following one predator-one prey system with prey-taxis

A1 $$\begin{aligned} {\left\{ \begin{array}{ll} u_{t}= d_1\Delta u+u\left( 1-u / K\right) -w f(u), & x\in \Omega ,\ t>0, \\ w_{t}=d_2\Delta w-\chi \nabla \cdot \left( w\nabla u\right) +w\left( \beta f(u)-\theta \right) , & x\in \Omega ,\ t>0, \\ \partial _{\nu } u=\partial _{\nu } w=0, & x\in \partial \Omega ,\ t>0, \end{array}\right. } \end{aligned}$$

where $\Omega \subset \mathbb {R}^n$ ($n\ge 1$) is a bounded domain with smooth boundary, $\partial _\nu :=\frac{\partial }{\partial \nu }$ and $\nu$ is the outward unit normal vector on $\partial \Omega$, *u* and *w* denote the densities of the prey and the predator species, respectively. The system (A1) is a special case of the system (1.3) without the invasive prey species. Parameters $d_1$ and $d_2$ are diffusion rates of the prey and the predator, respectively; $\chi \ge 0$ is the rate measuring the strength of prey-taxis; *K* is the carrying capacity for the prey; $\beta$ is the trophic efficiency. We consider the Holling type II functional response

A2 $$\begin{aligned} f(u)=\frac{\alpha u}{1+\alpha h u}, \end{aligned}$$

and the case of Holling type I functional response is discussed in Remark A.1. In (A2), $\alpha$ is the capture rate and *h* is the handling time. All the parameters above are positive except $\chi \ge 0$. We shall show that in the presence of prey-taxis, Turing instability is impossible in the system (A1) with (A2). More general prey-taxis models involving (A1) have been considered in Jin and Wang (2017, 2021), where no spatial patterns are observed.

Clearly, the system (A1) with (A2) has the constant steady states

A3 $$\begin{aligned} {\left\{ \begin{array}{ll} E_0, E_1, E_*, & \quad \text {if }\theta \in (0,\beta f(K)),\\ E_0, E_1, & \quad \text {if }\theta \in [\beta f(K),\infty ), \end{array}\right. } \end{aligned}$$

where $E_0=(0,0)$ is the extinction steady state, $E_1=(K,0)$ the predator-free constant steady state, and $E_*$ is the unique positive constant steady state with

$$\begin{aligned} E_*=\left( \frac{\theta }{\alpha (\beta -h \theta )},\frac{\beta (\beta f(K)-\theta )}{\alpha f(K) (\beta -h \theta )^2}\right) ,\quad \text {if }\theta \in (0,\beta f(K)). \end{aligned}$$

To investigate the linear stability of these constant steady states, we linearize the system (A1) at a constant steady state $E_s=(u_s,w_s)$ to get

$$\begin{aligned} \left\{ \begin{array}{llll} \Psi _{t}=\mathcal {M} \Delta \Psi +\mathcal {J} \Psi , & x \in \Omega , t>0, \\ \partial _{\nu } \Psi =0, & x \in \partial \Omega , t>0, \\ \Psi (x, 0)=\left( u_{0}-u_{s},w_{0}-w_{s}\right) ^{\mathcal {T}}, & x \in \Omega , \end{array} \right. \end{aligned}$$

where $\Psi (x, t)=\left( u-u_{s}, w-w_{s}\right) ^{\mathcal {T}}$ and $\partial _{\nu } \Psi (x, t)=\left( \partial _{\nu } u,\partial _{\nu } w\right) ^{\mathcal {T}}$ with $\mathcal {T}$ denoting the transpose, the two matrices $\mathcal {M}=\mathcal {M}(E_s)$ and $\mathcal {J}=\mathcal {J}(E_s)$ are given by

$$\begin{aligned} \mathcal {M}:= \left( \begin{array}{cc} d_1 & 0 \\ -\chi w_{s} & d_2 \end{array} \right) \quad \text {and}\quad \mathcal {J}&:= \left( \begin{array}{ccc} 1-\frac{2 u_s}{K_1}-w_s f_1'(u_s) & -f_1(u_s) \\ \beta _1 w_s f_1'(u_s)& \beta _{1} f_{1}(u_s)+\beta _{2} f_{2}(v_s)-\theta \\ \end{array} \right) . \end{aligned}$$

By similar arguments to those used in the derivation of (4.8), we obtain the characteristic equation

A4 $$\begin{aligned} {\rho _k^2}+A_1{\rho _k}+A_0=0, \end{aligned}$$

where

A5 $$\begin{aligned} {\left\{ \begin{array}{ll} A_1:={\mu _k} (d_1+d_2)-tr({\mathcal {J}}),\\ A_0:= d_1d_2 {\mu _k^2}+\Gamma {\mu _k}+Det(\mathcal J)\quad \text {with }\Gamma :=-(d_1J_{22}+d_2J_{11}+\chi w_s J_{12}). \end{array}\right. } \end{aligned}$$

Then the constant steady state $E_s$ is linearly stable if and only if $A_1, A_0>0$ for all $k\ge 0$.

### A.1 Stability analysis for the ODE (non-spatial) model

In the absence of spatial structure effects, the system (A1) becomes

A6 $$\begin{aligned} {\left\{ \begin{array}{ll} u_{t}= u\left( 1-u / K\right) -w f(u), & t>0, \\ w_{t}= w\left( \beta f(u)-\theta \right) , & t>0, \end{array}\right. } \end{aligned}$$

which has three possible equilibria given in (A3). The ODE system (A6) with the Holling type II functional response has been extensively studied, and we refer the readers to Hsu (2005); Yi et al. (2009) and the references therein. We have the following linear stability results for (A6) when *f*(*u*) takes the specific form (A2).

#### Lemma A.1

For the ODE system (A6) with (A2), we have the following stability results.

(i) $$E_0$$ is a saddle;
(ii) $E_1$ is linearly stable, marginally stable and linearly unstable for $\theta >\beta f(K)$, $\theta =\beta f(K)$ and $\theta \in (0,\beta f(K))$, respectively.
(iii) If $0<h\le \frac{1}{K\alpha }$, then $E_*$ is linearly stable for $\theta \in (0,\beta f(K))$; if $h> \frac{1}{K\alpha }$, then $E_*$ is linearly stable, marginally stable and linearly unstable for $\theta \in \left( \theta _0,\beta f(K)\right)$, $\theta =\theta _0$ and $\theta \in \left( 0, \theta _0\right)$, respectively. Here, $\theta _0:=\Big [\frac{\beta (\alpha h K-1)}{h (\alpha h K+1)}\Big ]_+$.

#### Proof

(i) Let $E_s=E_0$. Then the two eigenvalues of $\mathcal {J}$ are $-\theta$ and 1, which shows that $E_0$ is a saddle. (ii) Let $E_s=E_1$. Then the two eigenvalues of $\mathcal {J}$ are $-1$ and $\beta f(K)-\theta$, which proves the second result. (iii) Let $\theta \in (0,\beta f(K))$ and $E_s=E_*$. Then (A5) with $k=0$ and ${\mu _0}=0$ shows that

$$\begin{aligned} A_1=-tr({\mathcal {J}})=\frac{h \theta \left( \theta -\frac{\beta (\alpha h K-1)}{h (\alpha h K+1)}\right) }{\beta f(K) (\beta -h \theta )},\quad A_0= Det({\mathcal {J}})=\frac{\theta (\beta f(K)-\theta )}{\beta f(K)}>0. \end{aligned}$$

With the basic fact that $\theta<\beta f(K)=\frac{\beta \alpha K}{1+\alpha h K}<\frac{\beta }{h}$, we know that $sign(A_1)=sign\Big (\theta -\frac{\beta (\alpha h K-1)}{h (\alpha h K+1)}\Big )$. Then the third conclusion follows immediately. The proof is completed. $\square$

### A.2 Stability analysis for the spatial model

Turing instability means the constant steady state which is linearly stable in the non-spatial model becomes unstable in the presence of the spatial movement. In the case of $\alpha h K<1$ (which is used for the hypothesis (H4) in Jin and Wang (2017)), (Jin and Wang 2017, Theorem 1.3) obtained the global stability of $E_1$ for $\theta >\beta f(K)$, and the global stability of $E_*$ under certain parameter conditions (notably, Jin and Wang 2017 considered more general forms of functional responses and the prey growth functions including the case here). For the system (A1) with (A2) and general parameters, we next show that there is no Turing instability.

#### Lemma A.2

There is no instability driven by diffusion or prey-taxis in the spatial system (A1) with (A2).

#### Proof

Given Lemma A.1, we only need to investigate the stability of $E_1$ for $\theta >\beta f(K)$, and the stability of $E_*$ for $\theta \in (\theta _0, \beta f(K))$. We first let $E_s=E_1$, then the two roots of (A4) are $-1-d_1{\mu _k}<0$ and $-d_2{\mu _k}+\beta f(K)-\theta <0$ for $\theta >\beta f(K)$, which shows that $E_1$ is linearly stable. It remains to consider the stability of $E_*$ for $\theta \in (\theta _0,\beta f(K))$. Let $E_s=E_*$. Then $\Gamma$ given by (A5) satisfies

$$\begin{aligned} \Gamma =\frac{\theta \Big [\alpha h d_2(\beta -h \theta ) \Big (\theta -\frac{\beta (\alpha h K-1)}{h (\alpha h K+1)}\Big )+\beta \chi (\beta f(K)-\theta )\Big ]}{\alpha \beta f(K) (\beta -h \theta )^2}>0, \end{aligned}$$

where we have used the fact that $\frac{\beta (\alpha h K-1)}{h (\alpha h K+1)}\le \theta _0<\theta<\beta f(K)<\frac{\beta }{h}$ for $\theta \in (\theta _0,\beta f(K))$. Then (A5) along with $\Gamma ,-tr({\mathcal {J}}), Det({\mathcal {J}})>0$ implies $A_1,A_0>0$. Hence the two roots of (A4) have negative real parts and $E_*$ is linearly stable. This completes the proof. $\square$

#### Remark A.1

If the functional response *f*(*u*) is of Holling type I

A7 $$\begin{aligned} f(u)=\alpha u \end{aligned}$$

with a constant $\alpha >0$, then there is no Turing instability in the system (A1) with (A7). The proof parallels the Holling type II case but requires simpler computations and it is omitted for brevity.
